# PSGL-1 Blockade Induces Classical Activation of Human Tumor-associated Macrophages

**DOI:** 10.1158/2767-9764.CRC-22-0513

**Published:** 2023-10-26

**Authors:** Kevin Kauffman, Denise Manfra, Dominika Nowakowska, Mohammad Zafari, Phuong A. Nguyen, Ryan Phennicie, Elisabeth H. Vollmann, Brian O'Nuallain, Sara Basinski, Veronica Komoroski, Kate Rooney, Elizabeth K. Culyba, Joseph Wahle, Carola Ries, Michael Brehm, Steve Sazinsky, Igor Feldman, Tatiana I. Novobrantseva

**Affiliations:** 1 Verseau Therapeutics, Auburndale, Massachusetts.; 2 Dr. Carola Ries Consulting, Penzberg, Germany.; 3 University of Massachusetts Medical School, Worcester, Massachusetts.; 4 Currently employed by Moderna Therapeutics, Cambridge, Massachusetts.

## Abstract

The immune suppressive microenvironment is a major culprit for difficult-to-treat solid cancers. Particularly, inhibitory tumor-associated macrophages (TAM) define the resistant nature of the tumor milieu. To define tumor-enabling mechanisms of TAMs, we analyzed molecular clinical datasets correlating cell surface receptors with the TAM infiltrate. Though P-selectin glycoprotein ligand-1 (PSGL-1) is found on other immune cells and functions as an adhesion molecule, PSGL-1 is highly expressed on TAMs across multiple tumor types. siRNA-mediated knockdown and antibody-mediated inhibition revealed a role for PSGL-1 in maintaining an immune suppressed macrophage state. PSGL-1 knockdown or inhibition enhanced proinflammatory mediator release across assays and donors *in vitro*. In several syngeneic mouse models, PSGL-1 blockade alone and in combination with PD-1 blockade reduced tumor growth. Using a humanized tumor model, we observed the proinflammatory TAM switch following treatment with an anti-PSGL-1 antibody. In *ex vivo* patient-derived tumor cultures, a PSGL-1 blocking antibody increased expression of macrophage-derived proinflammatory cytokines, as well as IFNγ, indicative of T-cell activation. Our data demonstrate that PSGL-1 blockade reprograms TAMs, offering a new therapeutic avenue to patients not responding to T-cell immunotherapies, as well as patients with tumors devoid of T cells.

**Significance:**

This work is a significant and actionable advance, as it offers a novel approach to treating patients with cancer who do not respond to T-cell checkpoint inhibitors, as well as to patients with tumors lacking T-cell infiltration. We expect that this mechanism will be applicable in multiple indications characterized by infiltration of TAMs.

## Introduction

Checkpoint inhibitors targeting receptors expressed on tumor-infiltrating T cells, such as PD-1 and CTLA-4, have revolutionized immuno-oncology, resulting in durable clinical remissions in a subset of patients across multiple cancer indications. Unfortunately, CTLA-4 and PD-1/PD-L1 blockade as monotherapies have limited activity with objective response rates of approximately 10%–16% and 25%–40%, respectively, depending on the tumor type (1–4). T-cell checkpoint inhibitors rely on the presence of functional tumor antigen–specific cytotoxic T cells in the tumor microenvironment (TME) at the tumor core to enable their reactivation (5, 6); however, the majority of human tumors lacks such T-cell infiltration at the time when the disease presents itself clinically and is therefore referred to as “cold” (7). Such tumors are not amenable to T-cell checkpoint therapies. In most patients across multiple tumor types, tumor-associated macrophages (TAM) are a major constituent of the immune cell population and a high degree of TAM infiltration has been correlated with poor prognosis and tumor metastasis (8, 9). Macrophages possess remarkable functional heterogeneity, capable of existing on a multidimensional continuum from proinflammatory, classically-activated M1-like macrophages, to anti-inflammatory, alternatively-activated M2-like macrophages (10, 11). The suppressive M2-like phenotype supports tumor progression by blocking antitumor immunity and by producing anti-inflammatory and proangiogenic factors (4, 12, 13). In contrast, the proinflammatory macrophage induces antitumor immune responses, directly attacks tumor cells as well as creates chemokine gradients calling upon other immune cells to enter the TME and activate them via cell-cell contact and activating soluble mediators (14, 15).

P-selectin glycoprotein ligand-1 (PSGL-1) is a homodimeric, transmembrane, mucin-like receptor, and its fundamental role in leukocyte tethering and adhesion on activated endothelium via binding to P-selectin and other selectins has been known for a long time (16–18). Various posttranslational modifications of PSGL-1 (e.g., glycosylation, sialylation) control its selectin binding capacity (19). While resting T cells can also express PSGL-1, only activated effector T cells acquire the capacity to bind selectins via glycosylation of PSGL-1. Apart from its established role in migration, PSGL-1 has several other described functions in myeloid cells: for example, engagement of PSGL-1 via P-selectin binding increases the tolerogenic behavior of human dendritic cells *in vitro* (20), and plate-bound agonist anti-PSGL-1 antibody was reported to drive the differentiation of murine monocytes into functional dendritic cells (21). Recent studies reported selectin-independent PSGL-1 signaling activities that contributed to T-cell exhaustion (22). PSGL-1–deficient *Selplg^−^^/^^−^* mice were found to have stronger immune responses as evidenced by significantly decreased tumor burden in a syngeneic melanoma model and improved viral clearance with increased proinflammatory cytokine release and less exhausted T cells in a chronic viral infection model (23). Consistent with this role in T-cell exhaustion, PSGL-1 has recently been identified as an acid-sensitive ligand for VISTA in the low-pH environment of tumor beds (24). Selectin-independent PSGL-1 signaling functions resulting in mTOR activation were also reported in adherent macrophages that relied on cytoskeletal rearrangements (22). Taken together, the reports described above, as well as by others (25–27), implicate a possible role for PSGL-1 in cancer.

Although macrophages and PSGL-1 have both been independently studied in the context of cancer, to our knowledge the function of PSGL-1 on human TAMs has not been investigated. Because PSGL-1 is known to mediate immunoregulatory functions and to be expressed on multiple immune cell types, we aimed to study whether it has an immune-regulatory role on TAMs given that TAM markers, such as CD163 (12), correlate with PSGL-1 by mRNA expression across multiple tumor types in our analysis. We report here a systematic analysis of PSGL-1 function in macrophages from various sources, including those derived from *in vitro* differentiation, patient tumors, and reconstitution in a humanized mouse model. PSGL-1 blockade via an antagonistic antibody repolarized M2 macrophages *in vitro* and induced a broad proinflammatory immune response in multicellular *in vitro* assays and *ex vivo* primary fresh human tumor cultures. In addition, PSGL-1 blockade in syngeneic and humanized mouse tumor models reduced tumor growth and activated T cells in the TME. In assays with multiple cell types present, it is impossible to exclude that PSGL-1 gets activated in other cells in addition to macrophages. Similarly, in previous articles describing PSGL-1 functionality in *in vivo* and complex *in vitro* systems focusing on T-cell functionality, macrophage activation could not be excluded. Here, we demonstrate an unexpected activity of PSGL-1, via antibody blockade and siRNA silencing in monocultures of human macrophages, and believe that PSGL-1 is a novel immunosuppressive macrophage checkpoint with potential broad applications in immuno-oncology.

## Materials and Methods

### Generation of Anti-murine PSGL-1 mAbs

Chicken anti-murine PSGL-1 antibodies were generated by phage display screening of Fab libraries generated from chickens immunized with recombinant His-tagged murine PSGL-1. Total mRNA harvested from single-cell suspensions of spleen and bone marrow were used as templates for generating phage display Fab libraries. Phage display Fab libraries were screened for binding to recombinant murine PSGL-1 and 293T cells stably expressing murine PSGL-1, and enriched clones were confirmed for specific binding to murine PSGL-1. Anti-murine PSGL-1 Fabs were reformatted as chimeric murine IgG1 antibodies, produced recombinantly, and characterized for their ability to activate, *ex vivo*, lipopolysaccharide (LPS)-stimulated splenocytes isolated from BALB/c mice. One chimeric murine IgG1 antibody was selected for *in vivo* studies.

### Screening for Functionality of Mouse Surrogate Anti-PSGL-1 Antibodies

#### Splenocyte Preparation

To prepare mouse splenocytes, spleens were dissociated by using a syringe to grind the spleen against a pre-wet (PBS) 70-µm filter on top of a 50-mL Falcon tube. The filter was washed with 20 mL of PBS and tubes centrifuged at 300 × *g* for 5 minutes, supernatant decanted and pellet resuspended in 2 mL Ammonium-Chloride-Potassium (ACK) lysing buffer. Tubes were briefly vortexed and incubated for 5 minutes at room temperature. The cell suspension was neutralized by adding 20 mL of PBS buffer and transferred to a new 50 mL conical by pouring the suspension through a fresh 70-µm filter. Tubes were centrifuged at 300 × *g* for 5 minutes, supernatant decanted, and cells washed one additional time with PBS.

#### Testing PSGL-1 Surrogate Antibody Binding to Splenic Macrophages

Cell pellets containing splenic macrophages were resuspended in PBS at 500K cells per mL and 100 µL per well added in a 96-well V-bottom plate. Plates were centrifuged at 300 × *g* for 5 minutes, supernatant decanted, and cells washed with FACS buffer. Cell pellets were resuspended in mouse Fc block diluted 1:20 in FACS buffer for 15 minutes on ice. Plates were centrifuged and cells resuspended in 100 µL of FACS buffer; 2 µL of anti-mouse PSGL-1 antibodies were added to achieve a final concentration of 10 µg/mL. Cells were mixed with a pipet, incubated on ice for 15 minutes, and washed 2X with FACS buffer. Cells were resuspended to 100 µL with a mixture of the following antibodies: secondary mouse IgG1 (BioLegend, 406608), phenotyping antibodies CD45 (BioLegend, 103147), and CD11b (BioLegend, 101262), and a live/dead dye (eBioScience, 65086614) for 20 minutes on ice. The cells were then centrifuged and washed 2x with FACs buffer and acquired using an AttuneTM NxT Flow Cytometer using AttuneTM NxT Software (Version 4.2.0) for acquisition. Data were analyzed using FlowJo software version 10.7.1 (Becton Dickinson & Company). The binding, as indicated by mean fluorescence intensity (MFI), of the 08A12 was measured on macrophage populations were defined as CD45^+^ CD11b^+^ viable macrophages.

#### PSGL-1 Surrogate Antibody Functional Activity on Mouse Splenocytes

Splenocytes were resuspended at 2 × 10^6^ cells/mL in complete RPMI. A total of 100 µL of the cell suspension was added to a U-bottom plate. A total of 275 µL of 4X antibody (40 µg/mL) solution was prepared and 50 µL of each antibody was added to appropriate wells for a final concentration of 10 µg/mL, and the cells were incubated for 15 minutes at 37°C. A total of 50 µL of 4x LPS (120 ng/mL, InvivoGen, catalog no. tlrl-eblps) was added at 30 ng/mL final concentration and the cells were incubated for 24 hours. The supernatants were collected, and mediators were analyzed using a Th1/Th2 mouse 20-plex Luminex assay (EPX200-26090-901).

### Histology

A commercial antibody [rabbit mAb clone EPR22504-36 (Abcam, ab227836)] was selected to stain PSGL-1, based upon an initial screening of multiple mAbs (Applied Pathology Systems). Candidate PSGL-1 IHC mAbs were screened on a combination of positive and negative cell lines and healthy lung (positive control) and muscle (negative control) human tissue, then the selected mAb transferred to Flagship Biosciences for incorporation into a multiplex IHC panel for tumor microarray (TMA) screening. For CD3, clone LN10 was used (Leica, PA0553), for CD163 - clone 10D6 (Abcam, ab74604), for CD68 - clone KPI (Dako, M0814). PSGL-1 IHC staining protocols were optimized on the Leica Bond, all staining performance was done using standard protocols and evaluated by an MD pathologist at Flagship Biosciences.

H-score was calculated using the following formula, where H, M, L, and N denote the number of high, medium, low, and negative cells, respectively:



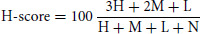



### Syngeneic Models

A PSGL-1 surrogate mouse antibody was evaluated in syngeneic mouse models. A total of 100 µL of 1 × 10^5^ MC38 cells, 5 × 10^5^ MB49 cells, 2 × 10^5^ EMT cells, or 8 × 10^6^ cells Sa1/N cells, all in 50% matrigel, were inoculated into the right flank of approximately 6-week-old female C57BL/6 mice from Charles River Labs (MC38, MB49), or AJ mice from JAX (Sa1/N). The MB49 cell line was licensed from EMD Millipore Corporation/Sigma. For MC38 and MB49, mice were randomized into different treatment arms when median tumor size reached 50–70 mm^3^ and the antibodies were dosed two times weekly at 10 mpk using 4–5 mL/kg volume. For Sa1/N, the mice were randomized on the basis of weight and the dosed as described above at day 1 postinoculation of the cells. The mice were dosed with anti-PD-1 (RMP1–14, BioXcell for MC38 and RMP1–14, ICHOR Bio for MB49 and Sa1/N), anti-mouse PSGL-1 antibody, or a combination of both antibodies. 2A3 rat IgG2a (BioXcell) and MOPC-21 mouse IgG1 (BioXcell) were used as the isotype controls for anti-PD-1 and anti-PSGL-1, respectively. See Supplementary Tables S2 and S3 for the flow cytometry staining panel.

### Humanized Mouse Model

A humanized NSG-SGM3-BLT mouse model of melanoma was set up as described in ref. 28. Briefly, male 10-week-old NSG-SGM3 purchased from Jackson were implanted with 1 mm^3^ fragments of human liver and thymus under the mouse kidney capsule. Two weeks later, the mice received 100 cGy gamma irradiation, followed by injection of 2 × 10^5^ CD34^+^ human hematopoietic progenitor cells (HSC) to reconstitute the human immune system. Six weeks after HSC injection, NSG-SGM3-BLT mice were inoculated subcutaneously with 5 million patient-derived xenograft melanoma (AV17.26) cells, and 2–4 weeks later human immune cell engraftment was verified. Mice with palpable (50–100 mm^3^) tumors were randomized into groups and treated with anti-PSGL-1 antibody at 10 mpk two times weekly for 3 weeks. Anti-PD-1 (Keytruda) was dosed at an initial dose of 10 mg/kg and all subsequent doses at 5 mg/kg—a regimen optimized by Brehm and colleagues in previous experiments (personal communication). Tumor volume was evaluated weekly by caliper. An IgG4 antibody with binding specificity to respiratory syncytial virus RSV was included as an isotype control for both anti-PD-1 and anti-PSGL-1. Tumors were harvested to evaluate immune cellular composition in the TME and spleens were harvested to evaluate composition of peripheral immune cell populations. See Supplementary Table S1 for the flow cytometry staining panel.

### *In Vitro* Generation of Monocyte-derived or Patient-derived Macrophages

Monocytes were isolated from whole blood of healthy donors by Ficoll separation and magnetic bead depletion using the RosetteSep Human Monocyte Enrichment Cocktail *(*Stemcell Technologies, catalog no. 15028). A total of 4 × 10^5^ monocytes per well were seeded in Iscove's modified Dulbecco's medium (IMDM) containing 10% FBS into 24-well plates. For monocyte differentiation, 50 ng/mL human MCSF (BioLegend) was added per well 24 hours after monocyte plating. On day 4, half the media was aspirated and 500 µL fresh media containing 50 ng/mL human MCSF was added per well. To polarize the differentiated M2-like cells to M2c macrophages, on day 6 the entire media content was aspirated and 1 mL of media (IMDM + 10% FBS) containing 50 ng/mL MCSF and 10 ng/mL IL10 (BioLegend) were added per well. On day 7, M2c macrophages were preincubated with anti-PSGL-1 antibody or isotype control antibody at a final concentration of 10 µg/mL for 30 minutes at 37°C, followed by activation with 100 ng/mL LPS (Invivogen) for an additional 24 hours. Cell culture supernatants were analyzed for secreted factors using ELISAs and Luminex.

### siRNA Design, Screening, and Formulation

siRNA lipid nanoparticles (siRNA-LNPs) were synthesized by mixing an aqueous phase containing siRNA in 10 mmol/L citrate buffer (pH 3) with an ethanolic solution containing ionizable lipid C12-200 (ref. 29; AxoLabs), 1,2-distearoyl-sn-glycero-3-phosphocholine (Avanti Polar Lipids), cholesterol (Sigma), and 1,2-dimyristoyl-sn-glycero-3-phosphoethanolamine-N-[methoxy(polyethylene glycol)-2000] (C14 PEG 2000, Avanti) at a 50:10:38.5:1.5 molar ratio and 9:1 total lipid:siRNA weight ratio. The aqueous and ethanol phases were mixed together at a 3:1 volume ratio in a microfluidic chip device using the NanoAssemblr (Precision Nanosystems). The resultant siRNA-LNPs were dialyzed overnight in a 20,000 molecular weight cutoff cassette against 1× PBS at room temperature. On average, siRNA-LNPs had a median diameter of approximately 60–70 nm as measured by nanoparticle tracking analysis (ZetaView, ParticleMetrix). The siRNA encapsulation efficiency (∼80%–90%) was determined using a modified Quant-iT Ribogreen Assay (Invitrogen) as described previously (30).

Purified human monocytes were isolated as described above and 400,000 cells were plated in IMDM 10% FBS in 24-well tissue culture plates. After 24 hours of adherence, media was removed and replaced with fresh IMDM 10% FBS with either 50 ng/mL GMCSF for the M1 conditions or 50 ng/mL MCSF for the M2 conditions. The monocytes were transfected by adding the siRNA-LNPs directly to the wells at a final concentration of 25 nmol/L. Two days later (day 3), a half media change was done by removing 250 µL of supernatant and adding 250 µL of fresh M1 or M2 media and siRNA LNPS. 2 days later (day 6) all supernatant was removed and replaced with 500 µL of M2c polarizing media (50 ng/mL MCSF + 10 ng/mL IL10). Two days later (day 8), the cells were harvested for branched DNA (bDNA) analysis to confirm target knockdown by a plate based qualitative branched DNA assay (QuantiGene Singleplex Assay Kit, Thermo Fisher Scientific). Cells were also analyzed via flow cytometry for functional markers of M1/M2 phenotype including CD163, CD206, and CD16. Day 8 supernatants were assessed for multiple cytokines and chemokines by Luminex bead-based multiplex cytokine array.

### *Ex Vivo* Patient Tumor Analysis

Fresh tumor tissue was placed in DMEM within 60 minutes of surgical resection, shipped overnight at +4°C, moved upon arrival to a tissue culture–treated petri dish containing 20 mL of cold Hank's Balanced Salt Solution (Gibco). After removing fat, fibrous, and necrotic areas, tumors were cut into small pieces of 2–4 mm and subsequently transferred into MACS enzyme mix and the tumors were further minced. The dissociation enzyme mix was prepared from a MACS tumor dissociation kit (Miltenyi Biotec) by adding 200 µL of Enzyme H, 100 µL of Enzyme R, and 25 µL Enzyme A to 4.7 mL of DMEM. Samples were vortexed and incubated at 37°C for 45 minutes to 1 hour. Digested tumors were filtered through 40 µm cell strainers and subsequently incubated in ice-cold DMEM supplemented with 8% FBS, 2% human serum, 100 IU/mL penicillin/streptomycin, 1 mmol/L Glutamax, 55 µmol/L 2-mercaptoethanol, 1X nonessential amino acids, 1 mmol/L sodium pyruvate, 100 µg/mL human recombinant IL2, 1X insulin/transferrin/selenium, and 4 ng/mL human MCSF to stop the enzymatic reaction. After centrifugation (5 minutes at 300 × *g*), cell pellets were resuspended in culture medium and the cells were counted. 5 × 10^5^ cells/mL were plated per well into 6-well plates and incubated with 10 µg/mL of each antibody (anti-PSGL-1, isotype control and pembrolizumab) for 48 hours. Supernatants from the dissociated tumor cultures were analyzed using a cytokine 25-plex human Luminex panel (Invitrogen) according to the manufacturer's instructions.

### Flow Cytometry

Humanized mouse model: Explanted tumors were mechanically dissociated by chopping and grinding tumor fragments through mesh screens followed by passing the suspension through cell strainers. A total of 1 mL of 5 × 10^8^ live single-cell suspension cells/mL were stained for viability using Fixable Viability Dye eFluor780 (eBioscience), followed by blocking unspecific antibody binding by incubating in 1 mL Fc-blocking buffer (human FcX; BioLegend) for 10 minutes. Following the blocking step, 100 µL of cells were combined with 100 µL of the lymphoid or myeloid staining cocktail and incubated for 1 hour on ice. The lymphoid staining cocktail contained CD3, CD4, CD8, CD16, CD20, CD25, CD45, CD45RA, CD56, MHCI, PD-1, and PSGL-1. The myeloid staining cocktail contained CD3, CD11b, CD14, CD16, CD33, CD45, CD86, CD163, CD206, CSF1R (CD115), HLA-DR (MHCII), PSGL-1, and VSIG4. Stained cells were resuspended in fixation buffer containing 0.32% paraformaldehyde (Thermo Fisher Scientific). Fixed samples were acquired using an AttuneTM NxT Flow Cytometer using AttuneTM NxT Software (Version 4.2.0) for acquisition. Data were analyzed using FlowJo software version 10.7.1 (Becton Dickinson & Company). Distinct macrophage populations were defined as CD45^+^ CD33^+^ or CD14^+^CD11b^+^ gated from CD45^+^ viable cells (Supplementary Fig. S9). Macrophage populations were then analyzed for the presence of M1 phenotypic markers (MHCII and CD86) or M2 phenotypic markers (CD163 or CD206).

### Human Staphylococcal Enterotoxin B Assay

Peripheral blood mononuclear cells (PBMC) were isolated from commercially obtained, anticoagulant treated blood preparation Leukopak by Ficoll gradient centrifugation. A total of 2 × 10^5^ cells were added to each well of a 96-well round bottom tissue culture plate. To determine the concentration of Staphylococcal enterotoxin B (SEB) allowing suboptimal stimulation of PBMCs, SEB was titrated from 1.875 µg/mL to 0.3 ng/mL using a 3-fold dilution and incubated with PBMCs from 2 donors and incubated at 37^°^C for 4 days. On day 4, the plates were centrifuged and the culture supernatants were collected and assayed for secreted IL2 by ELISA. On the basis of this data, 0.25 µg/mL was determined as the suboptimal concentration for this lot of SEB. 0.25 µg/mL SEB was used in subsequent assays with anti-PSGL-1 or isotype control antibody. 10 µg/mL of antibody was preincubated with the cells for 15 minutes before adding the SEB. After 4 days, cell culture supernatants were analyzed for secreted factors using ELISAs and Luminex. An IgG4 antibody with binding specificity to respiratory syncytial virus RSV was included as an isotype control.

### Protein Analysis

Cell culture supernatants were assessed using a Cytokine 11-plex human Luminex panel (Invitrogen) and the Luminex instrument according to the manufacturer's recommendation. IL2 and CCL4 concentration in cell supernatants was determined by specific ELISAs: Human IL2 beta DuoSet ELISA (BioLegend) and Human CCL4/MIP-1β DuoSet ELISA (R&D Systems) and performed according to the manufacturer's recommendation.

### Mixed Lymphocyte Reaction

On day 1, monocytes were isolated as described above, adjusted to 500,000 cells/mL in IMDM + 10% FBS, and 100 µL (50,000 cells) was added per well of a 96-well flat-bottom plate. A total of 24 hours later, there was a full media change with 200 µL of M1 (IMDM + 10% FBS + 50 ng/mL GMCSF) or 100 µL of M0 media (IMDM + 10% FBS + 50 ng/mL MCSF) followed by addition of 100 µL of M0 media with 20 µg/mL mAb (final concentration = 10 µg/mL). There was a half media change with 100 µL of M1 or 100 µL of M0 media containing 10 µg/mL mAb (final concentration = 10 µg/mL) on day 5. On day 7, there was a half media change with 100 µL of 2x activation media (IMDM + 10% FBS + 50 ng/mL GMCSF + 40 ng/mL IFNg + 200 ng/mL LPS) for M1 cells. To the M0 wells, 100 µL of media was removed from each well, and replaced with 100 µL of M0 media containing 20 µg/mL mAbs (final concentration = 10 µg/mL). On day 9, T cells were isolated from allogeneic donor-derived PBMCs using a Stem Cell total CD3-negative isolation kit (EasySep Human T Cell Isolation Kit | STEMCELL Technologies). Cells were washed one time with 200 µL of warm T-cell media (RMPI1640 containing 10% FBS, 1x NEAA, 1 mmol/L sodium pyruvate, 10 mmol/L HEPES and freshly added 55 µmol/L 2-mercaptoethanol) and resuspended at 1 million cells/mL. A total of 100 µL of T cells were added per well. The mixed lymphocyte reaction (MLR) was incubated for 4 days and on day 13, supernatant was collected for Luminex analysis of mediators, and the cells were processed for flow cytometry.

### Computational and Statistical Methods

Published single-cell RNA sequencing (scRNA-seq) data (31) were downloaded and analyzed using the BioTuring Browser software (https://bioturing.com/).

The Cancer Genome Atlas (TCGA) data were displayed using the Matlab software (https://www.mathworks.com/). Matlab was used to compute the Spearman correlation coefficients and *P* values.

Statistical significance for comparisons between treatment groups or conditions was assessed using a two-way ANOVA (or mixed-effect models when some datapoints were missing), Student *t* test, and Kolmogorov–Smirnov test in the Graph Prism software depending on experimental design. Exact tests used in each case are noted in the respective figure legends.

The data generated in this study are available upon request from the corresponding authors.

## Results

### PSGL-1 is Highly Expressed on TAMs

**FIGURE 1:**
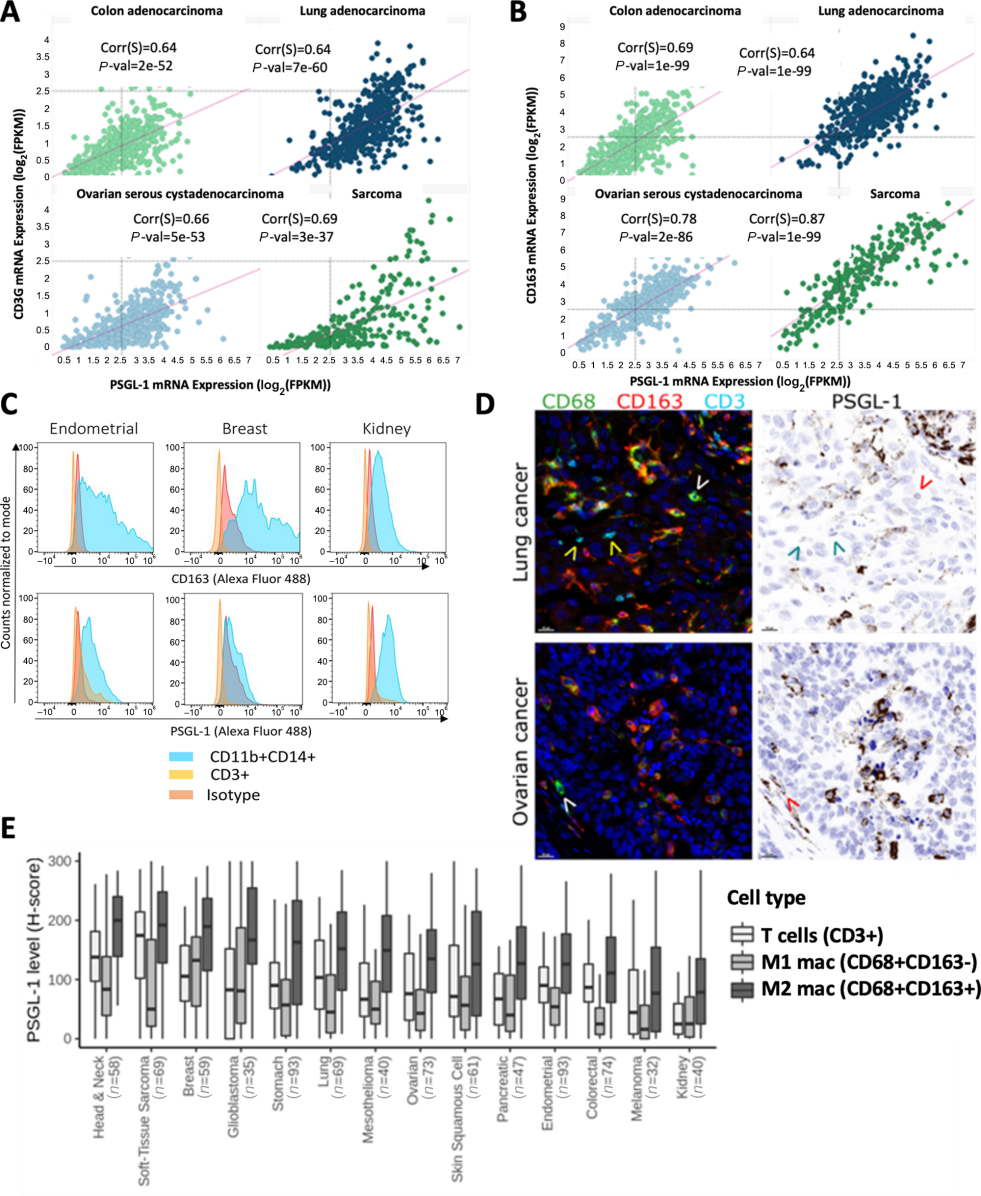
PSGL-1 expression is enriched in TAMs within human tumors. Correlation between mRNA levels of PSGL-1 and a T-cell marker (CD3G; **A**) or a macrophage marker (CD163; **B**) in selected tumor indications from TCGA dataset (ref. 32; Spearman rank correlations and *P* values). **C,** Cell surface expression of CD163 (top) and PSGL-1 (bottom) on CD11b^+^CD14^+^ macrophages and CD3^+^ T cells in various tumor tissues by flow cytometry. **D,** Colocalization of PSGL-1 protein with CD68^+^CD163^+^ macrophages in tumor tissue by fluorescent multiplex IHC. Note very low or no PSGL-1 staining on CD68^+^CD163^−^ macrophages (white/red arrow heads) and T cells (yellow/cyan arrow heads). Representative images from lung and ovarian TMAs. CD68, CD163, and CD3 are shown in pseudocolored fluorescence images; PSGL-1 is shown in a simulated DAB/hematoxylin image. Scale bar: 20 µm. **E,** Quantification of PSGL-1 expression on “M1” (CD68^+^CD163^−^) macrophages, “M2” (CD68^+^CD163^+^) macrophages and T cells in 14 TMAs each representing a different indication (*n* = number of unique patient tumor samples per indication). H-score is a semiquantitative measure of signal intensity ranging from 0 to 300.

By analyzing PSGL-1 mRNA (*SELPLG* gene) expression in a published scRNA-seq dataset comprising tumor derived and peripheral immune populations from 7 patients with non–small cell lung cancer (31), we found *SELPLG* is broadly expressed across immune cell populations within the TME (Supplementary Fig. S1). The gene is expressed in both lymphoid and myeloid cells. In the TME, the myeloid subsets expressing *SELPLG* are dominated by suppressive myeloid populations such as TAMs and myeloid-derived suppressor cells. This finding prompted us to extend our analysis to approximately 10,000 human tumors with bulk mRNA profiling by RNA-sequencing spanning 31 solid cancer types that are captured in TCGA dataset (32). In this dataset, we consistently found *SELPLG* expression in a diverse array of human tumor types. The highest *SELPLG* expressing tumor types included pancreas and lung adenocarcinomas, sarcoma, glioblastoma, and mesothelioma, which are tumor types known for an abundant TAM infiltrate correlating with poor survival (Supplementary Fig. S2; ref. 33). Although PSGL-1 has been reported to be an immune checkpoint regulator on T cells (20, 21), *SELPLG* mRNA expression correlates better with a TAM marker *CD163* than with CD3 gene expression in certain human tumor types (Spearman correlation coefficients, respectively): corr = 0.87, pval = 1e-99 vs. corr = 0.69, pval = 3e-37 in sarcoma; corr = 0.78, pval = 2e-86 vs. corr = 0.66, pval = 5e-53 in ovarian cancer; corr = 0.69, pval = 1e-99 vs. corr = 0.64, pval = 2e-52 in colon cancer; corr = 0.64, pval = 1e-99 vs. corr = 0.64, pval = 7e-60 in lung adenocarcinoma (Fig. 1A and B), implying that despite its broad expression pattern, much of *SELPLG* expressed in the TME of those tumor types is TAM-derived. This led us to further investigate the biological function of PSGL-1 on TAMs. While we focused our investigation on TAMs, we cannot exclude PSGL-1’s role on other cell types, such as neutrophils.

To validate the association of PSGL-1 and CD163 expression predicted from bulk and scRNA-seq data from human tumors, dissociated primary human tumors were analyzed by flow cytometry (Fig. 1C). In three individual tumors from the endometrium, breast, and kidney, CD14^+^/CD11b^+^ TAMs expressed both CD163 and PSGL-1 on their surface. Both CD163 and PSGL-1 are present on nearly every CD11b^+^ cell in each sample, implying coexpression. In contrast, PSGL-1 was more weakly expressed on the surface of tumor-associated CD3^+^ T cells as measured by MFI (Fig. 1C).

We further performed multiplex IHC on human TMAs. CD68 was used as a pan-macrophage marker, CD163 as a marker for M2-like protumorigenic macrophages, and T cells were identified using CD3. PSGL-1 was highly enriched on CD68^+^CD163^+^ M2-like macrophages but was notably lower or absent on M1-like (CD68^+^CD163^−^) macrophages and T cells (Fig. 1D). Quantification of PSGL-1 expression on these three cell types across 14 different indications (∼30 to 100 patient tumors per indication) showed that PSGL-1 is consistently enriched on M2-like macrophages (Fig. 1E). Taken together, strong coexpression of PSGL-1 and CD163 in the tumor identifies PSGL-1 as a possible checkpoint on suppressive TAMs.

### PSGL-1 Inhibition on Suppressive Macrophages Results in Proinflammatory Reprogramming

**FIGURE 2:**
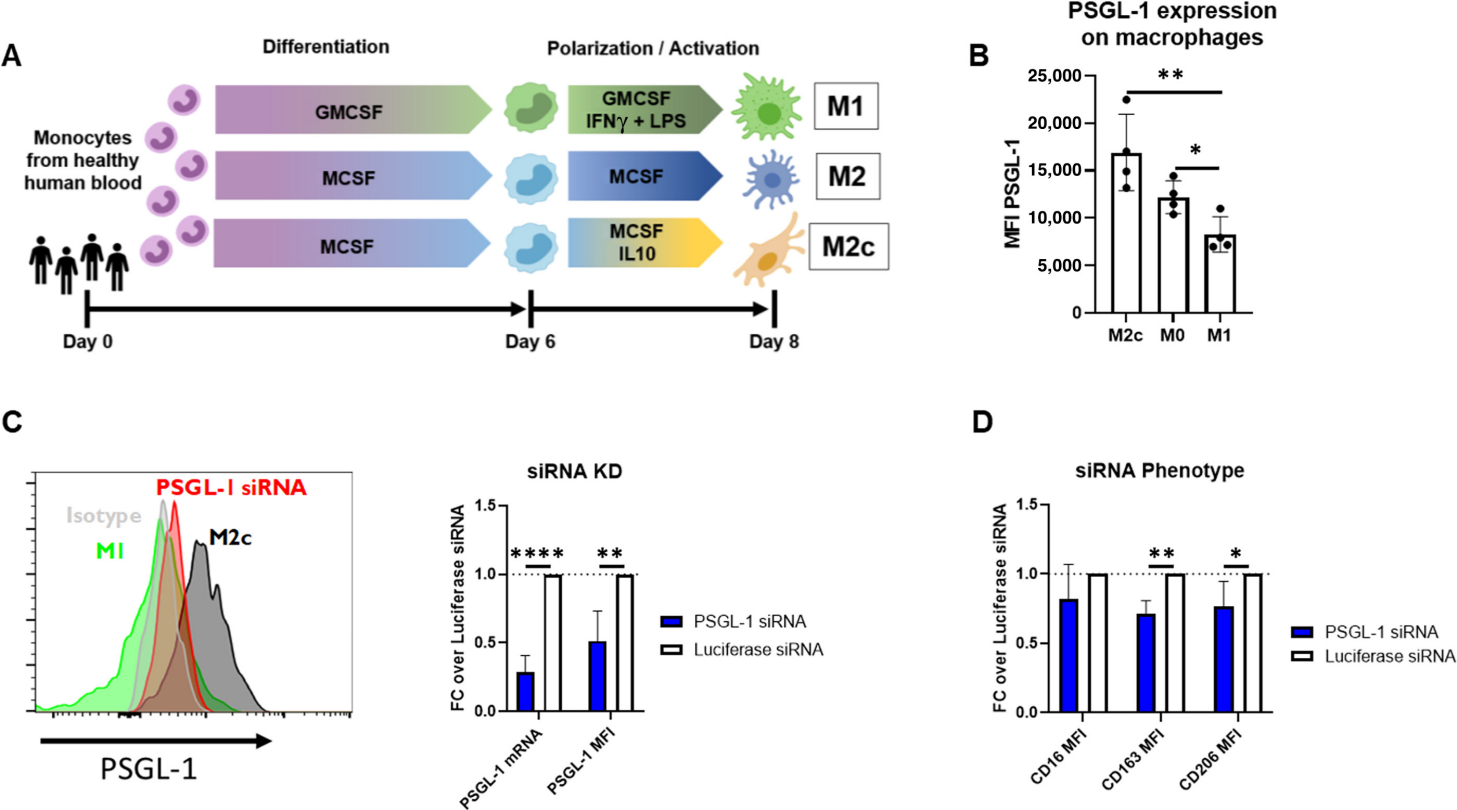
PSGL-1 is expressed on human M2-like macrophages. **A,** Schematic of M1, M0, and M2c macrophage differentiation and polarization conditions using human peripheral blood monocytes. **B,** PSGL-1 expression on M0, M2c, and M1-like macrophages. Data are presented as an average MFI across 4 donors and is representative of six experiments. **C,** siRNA knockdown of PSGL-1 in M2c macrophages transfected with 25 nmol/L PSGL-1 siRNA LNPs on days 1 and 3 during the differentiation and polarization period. PSGL-1 mRNA and protein levels were measured on day 8 by branched chain DNA analysis and flow cytometry, respectively (*n* = 4 donors). **D,** Fold change of M2c markers CD16, CD163, and CD206 as determined by flow cytometry in M2c macrophages treated with siRNA LNPs relative to those treated with Luciferase siRNA. Significance measured using Student *t* test in Graph Prism software for each pair of phenotypic macrophage conditions (B) or siRNA knockdown and phenotypic change (C, D).

CD163^+^ TAMs are associated with a suppressive, M2-like phenotype (12). Because macrophages can exist in a continuum of states ranging from anti-inflammatory to proinflammatory, we next investigated whether CD163/PSGL-1 coexpression could be replicated *in vitro* and whether PSGL-1 expression was critical to maintain a suppressive macrophage phenotype and function. Human blood monocytes were differentiated into proinflammatory M1 macrophages, suppressive M0/M2 macrophages, or highly suppressive M2c macrophages (Fig. 2A; ref. 10), and the resulting phenotypes were confirmed by measuring expression of a classical M2 marker, CD163, and classical M1 markers, CD86 and MHC-II, by flow cytometry (Supplementary Fig. S3). As shown in Fig. 2B, PSGL-1 protein is most highly expressed on suppressive M2c macrophages and least expressed on proinflammatory M1 macrophages. Markedly higher PSGL-1 expression on suppressive monocyte-derived macrophages *in vitro* across multiple donors is consistent with the previous finding that PSGL-1 is highly expressed on suppressive TAMs in primary human tumors.

**FIGURE 3:**
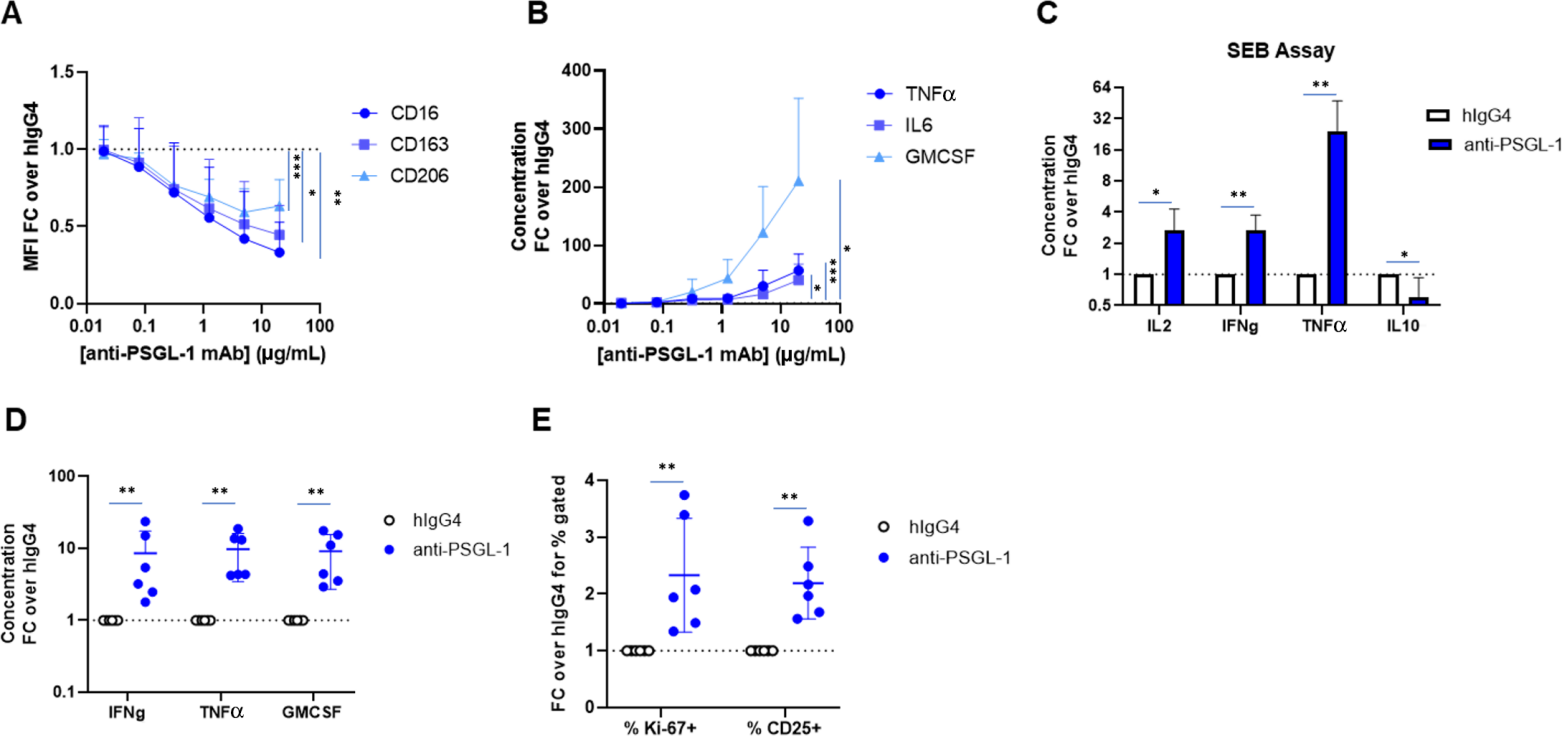
Anti-PSGL-1 repolarizes macrophages and leads to a proinflammatory response. **A,** Fold change of M2 markers CD163, CD206, and CD16 measured by flow cytometry on LPS stimulated M2c macrophages treated with anti-PSGL-1 relative to IgG4 isotype control (*n* = 4 donors). **B,** Fold change of secreted proteins TNFα, IL6, and GMCSF measured by Luminex in the supernatants from LPS stimulated M2c macrophages treated with anti-PSGL-1 relative to IgG4 isotype control (*n* = 4 donors). See Supplementary Fig. S5 for absolute levels in pg/mL for reference. For both A and B, antibodies were added at various concentrations to M2c on day 7 of the polarization process for 30 minutes followed by the addition of 100 ng/mL LPS for 24 hours. **C,** Fold change of secreted proinflammatory proteins IL2, TNFα, and IFNγ measured in the supernatants from a multicellular PBMC-SEB assay treated with anti-PSGL-1 relative to the IgG4 isotype control treatment. PBMCs were sequentially treated with anti-PSGL-1 at 10 ug/mL for 30 minutes followed by SEB for 3 days. On day 3, the supernatants were collected, and secreted proteins were measured in triplicate by a Luminex custom 10-plex. (*n* = 6 donors). **D,** Fold change of secreted cytokines from an MLR assay. Monocytes were treated with anti-PSGL-1 throughout the 9-day differentiation and polarization of M0 macrophages and then coincubated with allogeneic T cells for 4 days. On day 13, Luminex was used to measure secreted mediators in the supernatants (D) and flow cytometry was used to measure T cells proliferation and activation (**E**). Significance was measured using mixed-effect models by concentration and treatment for each marker using Graph Prism software in A and B. Significance was measured using Kolmogorov–Smirnov test for each analyte using Graph Prism software in C, D, and E (given the large variance of fold changes across samples for the same analyte, the nonparametric test was more sensitive than the *t* test for most comparisons; however, the *t* test produced statistically significant results for all the comparisons as well).

To probe the function of PSGL-1 on suppressive macrophages, we performed siRNA-mediated silencing (34) of PSGL-1 on *in vitro-*derived M2c macrophages. Knockdown of PSGL-1 receptor at about 50% on protein level (Fig. 2C) resulted in the reduction of classical M2-associated markers CD16, CD163, and CD206 by flow cytometry (Fig. 2D), suggesting that PSGL-1 may be an immunomodulatory receptor which plays a role in supporting the M2-polarization of macrophages. Similarly, addition of an anti-PSGL-1 mAb (“anti-PSGL-1”; Supplementary Fig. S4; ref. 35) to M2c macrophages prior to LPS activation reduced expression of CD16, CD163, and CD206 in a dose-dependent fashion (Fig. 3A). Thus, PSGL-1 inhibition by two different modalities (siRNA and mAb) phenotypically repolarizes M2c macrophages as measured by expression of M2-associated receptors.

We next investigated whether PSGL-1-inhibited macrophages became functionally more proinflammatory as well. Anti-PSGL-1 mAb induced M2c macrophages stimulated with LPS to produce significantly more proinflammatory cytokines TNFα, IL6, and GMCSF than those treated with an isotype control in a dose-dependent fashion (Fig. 3B; Supplementary Fig. S5). Antibody-mediated blockade of PSGL-1 on Toll-like receptor (TLR)-stimulated primary human monocytes and various human monocytic tumor cell lines also resulted in increased proinflammatory cytokine and chemokine production (Supplementary Fig. S6). Together, these data suggests PSGL-1 blockade both phenotypically and functionally repolarizes suppressive human macrophages *in vitro* toward a more inflammatory state.

### PSGL-1 Blockade in a Multicellular Context Increases Functional T-cell Activation

After establishing that in purified macrophage monocultures PSGL-1 inhibition leads to proinflammatory reprogramming, we wanted to test whether the same happens in multicellular cocultures. The stimulation of PBMCs with SEB, which crosslinks MHC-II expressed on antigen-presenting cells with the T-cell receptor on T cells, has been used to screen the effect of antibodies on immune cell activation in PBMCs (36). Anti-PSGL-1 mAb treatment in this assay led to statistically significant increases of multiple proinflammatory cytokines in the supernatant and a reduction of the anti-inflammatory cytokine IL10 across multiple donors (Fig. 3C). This cytokine signature, and particularly the increase in T cell–associated cytokines IL2 and IFNγ, indicates increased T-cell activation and proliferation in response to anti-PSGL-1 mAb. Enhancement of SEB-mediated stimulation of PBMCs is consistent with the hypothesis that PSGL-1 blockade on TAMs may lead to a coordinated antitumor immune response. Given that PSGL-1 can be expressed on multiple cell types in this assay, we do not know which cell type is driving the PSGL-1 inhibition effect in SEB-stimulated PBMCs.

In a second model, a MLR assay, suppressive M2 macrophages were treated with anti-PSGL-1 mAb and washed prior to the addition of allogeneic T cells to restrict PSGL-1 inhibition to M2 like macrophages. After 4 days of incubation, significantly higher levels of proinflammatory cytokines were observed in the supernatant (Fig. 3D), and a higher proportion of T cells were proliferating (as defined by Ki-67^+^ cell fractions) and activated as evidenced by CD25 positivity when macrophages were treated with anti-PSGL-1 mAb compared with isotype (Fig. 3E). Because the T cells were not treated with anti-PSGL-1 mAb in this assay, it follows that suppressive macrophage repolarization via PSGL-1 blockade directly translates to increased stimulation of cocultured allogeneic T cells. The MLR assay illustrates that anti-PSGL-1 mAb treatment of macrophages leads to increased activation of T cells and a coordinated proinflammatory immune response, supporting the hypothesis that PSGL-1 blockade on TAMs may lead to a multicellular antitumor response.

### PSGL-1 Blockade Results in Suppression of Tumor Growth in Syngeneic Mouse Models as Monotherapy or Combination Therapy with Anti-PD-1

**FIGURE 4:**
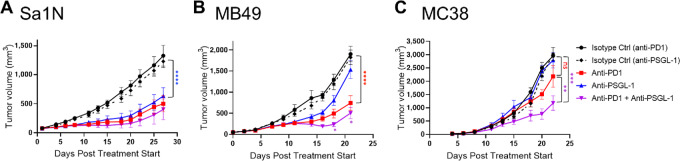
Antitumor effects of a surrogate murine anti-PSGL-1 in syngeneic mouse models. Mean tumor volumes ± SEM in mice treated with anti-PSGL-1, anti-PD-1, anti-PSGL-1 in combination with anti-PD-1 or their corresponding isotype controls (*n* = 8–10 mice per group) from the Sa1N sarcoma (**A**), MB49 bladder cancer (**B**), or MC38 colon carcinoma (**C**) models. Two-way ANOVA in Graph Prism software was used to assess significance of difference between entire treatment groups and two-way ANOVA with multiple comparisons was used to assess significance of difference for individual timepoints when the entire group comparison was not significant. “ns” stands for no significance.

To determine whether repolarization of macrophages and its downstream effects translate into *in vivo* activity, a surrogate mouse anti-PSGL-1 antibody was evaluated in syngeneic mouse models. The surrogate mouse antibody, clone 08A12, was selected because of its specificity for mouse PSGL-1 and its ability to induce a proinflammatory response in mouse splenocytes (Supplementary Fig. S7). Anti-mouse PSGL-1 was efficacious in several models evaluated, including the Sa1N sarcoma, MB49 bladder cancer, MC38 colon carcinoma (Fig. 4) models. Reduced tumor growth was observed for both monotherapy and combination therapy with anti-PD-1 and the degree of tumor growth inhibition was model dependent. PSGL-1 monotherapy significantly reduced tumor growth in Sa1N and MB49 models (*P* < 0.0001 and *P* = 0.0005 compared with isotype control groups, respectively; Fig. 4A and B). Although there was no PSGL-1 monotherapy effect observed in the MC38 model, there was significant combination effect when compared with PD-1 monotherapy (*P* = 0.0005; Fig. 4C). Such combination effect was also observed for the MB49 model (*P =* 0.02). These data support targeting PSGL-1 alone or in combination with PD-1 blockade as a promising therapeutic approach across multiple cancer types.

### PSGL-1 Blockade Switches TAMs to a More Proinflammatory Phenotype and Promotes T-cell Tumor Infiltration in a Humanized Mouse Model of Cancer

**FIGURE 5:**
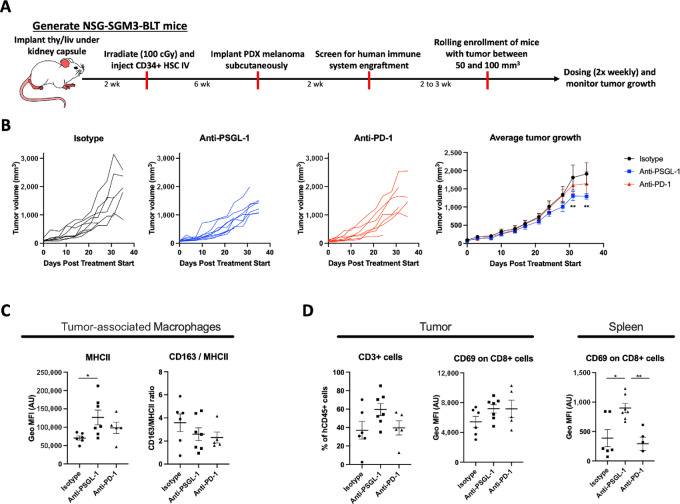
Antitumor effects of anti-PSGL-1 in a humanized mouse model. **A,** Schematic of the NGS-SGM3-BLT humanized mouse melanoma model. **B,** Tumor volume of melanoma tumors in NGS-SGM3-BLT mice treated with anti-PSGL-1 or an Isotype control (*n* = 8–9 mice per group). **C,** MFI of MHCII and the ratio of the MFI of CD163 to MHCII was assessed on CD11b^+^CD14^+^ macrophages within the tumor measured by flow cytometry. **D,** The percent of total CD3^+^ T cells in the tumor and the activation status of T cells in both the tumor and spleen measured by the MFI CD69 on CD3^+^ T cells. Two-way ANOVA with multiple comparisons in Graph Prism software was used to assess significance in B and a one-way ANOVA in Graph Prism was used to assess significance in C and D.

To translate our findings on macrophage activation and T-cell crosstalk induced by PSGL-1 blockade to an *in vivo* setting, we analyzed tumor growth and profiled tumor associated and peripheral immune cells in a patient-derived melanoma model (AV17.26) in humanized mice. We used NSG-SGM3-BLT mice which are on a NSG background, express transgenes for human stem cell factor, GMCSF and IL3 to support myeloid cell differentiation from CD34^+^ hematopoietic progenitors, and have transplanted liver and thymic tissue to provide more human like maturation environment for T cells (37). These mice were shown to have an improved engraftment and function of human CD33^+^ myeloid cells compared with NSG mice and provide an excellent tool for studying the role of human macrophage modulation in an *in vivo* disease setting (37). Briefly, NSG-SGM3-BLT mice were screened for human CD45^+^ cell engraftment at 8–10 weeks after CD34^+^ engraftment (Fig. 5A) and inoculated subcutaneously with a human melanoma cell line. The AV17.26 model was selected because it is known to facilitate human TAM infiltration, most likely due to its expression of human CSF1. Once tumors reached a size of 50–150 mm^3^, mice were randomized into three treatment groups: isotype control, anti-human PSGL-1 mAb (described in the *in vitro* studies above), or anti-human PD-1 mAb (Keytruda). Blockade of PSGL-1 resulted in significantly reduced tumor growth, whereas PD-1 blockade did not significantly affect tumor growth (Fig. 5B).

When tumor tissues were immunophenotyped, we found that CD11b^+^/CD14^+^ macrophages in anti-PSGL-1-treated mice had a 1.8-fold upregulation of MHC-II M1 marker expression (Fig. 5C). To quantify macrophage phenotype repolarization, we analyzed the ratio of CD163 to MHC-II expression by flow cytometry. The CD163/MHC-II ratio decreased 1.4-fold with PSGL-1 blockade (Fig. 5C), demonstrating a reduction in protumorigenic M2 (CD163) marker expression with respect to antitumorigenic (MHC-II) M1 marker expression. We also found that PSGL-1 blockade resulted in a 1.6-fold higher frequency of tumor-infiltrating CD3^+^ cells and 30% higher expression of CD69 on intratumoral CD8^+^ T cells, indicating increased activation of these cells (Fig. 5D). A similar increase in CD69 expression on CD8^+^ T cells was observed on peripheral spleen cells (Fig. 5D). Overall, PSGL-1 inhibition was shown to have an antitumor effect in this humanized mouse model, mediated in part by repolarizing tolerogenic macrophage phenotype towards a more immunostimulatory one, and thereby promoting higher T-cell infiltration and activation.

### PSGL-1 Blockade Increases Proinflammatory Immune Response in *ex Vivo* Tumor Cultures, Including PD-1 Blockade Nonresponsive Tumors

**FIGURE 6:**
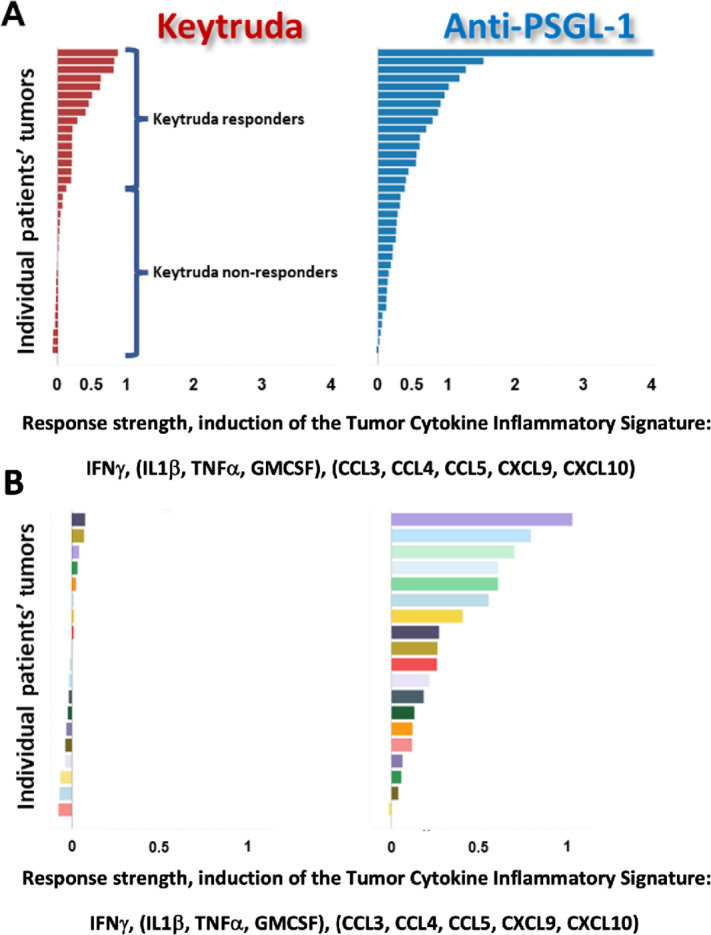
Anti-PSGL-1 monotherapy induces a secretion of proinflammatory cytokine signature in both Keytruda responders and Keytruda nonresponders in fresh tumor cultures. **A,** Waterfall plots of the Tumor Cytokine Inflammatory Signature value induced in individual tumors by Keytruda or the anti-PSGL-1 antibody over the isotype control (fold change of treatment over control). Tumors are ranked by the strength of induction by either Keytruda (red bars) or the anti-PSGL-1 antibody (blue bars). **B,** Nineteen tumors with the weakest response to Keytruda were designated as Keytruda nonresponders. In that subset of tumors, the anti-PSGL-1 antibody shows induction of the Tumor Cytokine Inflammatory Signature across many individual tumors (colors represent individual tumors and are the same in both treatment arms but ranked differently according to strength of response in each treatment arms).

Because humanized mice do not contain a fully functional complement of differentiated human immune cells (37, 38, 39), we extended our studies to cancer patient–derived tumors. These tumors come from surgical tumor removal with all the naturally infiltrating immune cells from the patient's immune system. Hence, we investigated whether PSGL-1 blockade could repolarize primary TAMs *ex vivo* and subsequently induce a proinflammatory response despite the presence of tumor cells and other TME-derived immune suppressive cells and factors. We aimed to analyze a broad spectrum of cancer types to cover the continuum of what is currently considered immunogenic and nonimmunogenic tumor types. Thirty-six resected human tumors representing five tumor types (ovary, uterus, kidney, omentum, and lung) were dissociated into single cell suspensions and cultured separately in the presence of anti-PSGL-1 mAb or controls. In each tumor, we surveyed changes induced by experimental treatment compared with a control arm for a broad spectrum of known soluble mediators of immune response. Induction of cytokines and chemokines detected in the tumor culture supernatant was quantified and grouped into three broad categories: (i) effector T cell–derived cytokine: IFNγ, (ii) myeloid-associated proinflammatory cytokines: TNFα, IL1-β and GMCSF, and (iii) chemokines involved in immune cell recruitment: CCL3, CCL4, CCL5, CXCL9, and CXCL10. For each treatment group, the Tumor Cytokine Inflammatory Signature was calculated as an unweighted mean across all analyte levels in all three categories. The treatment effect was assessed as a fold change of the treatment induction over the isotype control. Previously, in *ex vivo* tumor cultures, 30%–50% response in one of the analytes had been shown to correlate with clinical response (40, 41). We have used a more stringent criteria of 50% induction in the total Tumor Cytokine Inflammatory Signature value as the measurement of response to a treatment for a given tumor. Anti-PSGL-1 mAb produced a strong proinflammatory immune response across multiple tumors and tumor types by increasing cytokines involved in TME and T-cell activation as well as chemokines promoting immune cell infiltration (Fig. 6; Supplementary Fig. S8). Moreover, PSGL-1 blockade demonstrated an equal or greater total inflammatory response amplitude when compared with anti-PD-1 mAb (pembrolizumab). Comparison to PD-1 inhibition provides comparison to the level of modulation that was previously shown to translate into clinically meaningful changes from a drug approved in multiple cancer indications (42). More specifically, 14 out of 36 tumors responded to the PSGL-1 blockade while only 5 tumors responded to the anti-PD-1 treatment. Perhaps even more significantly, when we focused on tumor tissues least responding to PD-1 inhibition in the dish—19 tumors with less than 10% of induction in the Tumor Cytokine Inflammatory Signature value, which could be taken as representing the most significant clinical need not addressed by the current PD-1/L1 therapies, 15 tumors showed some level of response above that of PD-1, with 6 out of 19 tumors showing more than 50% of signature value induction.

## Discussion

Established tumors successfully escape the surveillance of the immune system and manage to hijack innate immune cells to promote tumor growth (43, 44). The molecular mechanisms underlying these processes remain largely elusive, albeit they provide potential therapeutic targets for treating checkpoint inhibitor resistant tumors. This study establishes PSGL-1 as a positive regulator of immunosuppressive macrophages. Antibody-mediated PSGL-1 blockade increased the proinflammatory response of human macrophages in isolation as well as in complex model systems such as patient-derived tumor cultures and in syngeneic murine models as well as a humanized mouse melanoma model.

While PSGL-1 is a well-known adhesion molecule involved in leukocyte trafficking, recent evidence for a signaling function has emerged (22, 45). Notably, PSGL-1 deficiency prevented T-cell exhaustion and improved viral clearance independent of selectin binding (22). Furthermore, *Selplg^−^^/^^−^* mice are characterized by developing spontaneous autoimmune disease or exacerbated experimental autoimmunity that is associated with impaired regulatory T cell (Treg) function (46, 47). Lack of PSGL-1 expression resulted in less tolerogenic dendritic cells displaying higher MHC class II expression (20). Conversely, activation of PSGL-1 via P-selectin binding induced tolerogenic dendritic cells that generated more Tregs (20). The molecular selectin-dependent and independent PSGL-1 receptor signaling that conveys these immunosuppressive functions is not yet entirely understood. Recently, VISTA was identified as a novel ligand and reported to mediate immunosuppressive T-cell effects via activation of the PSGL-1 transmembrane receptor (24). It is currently unclear whether additional, yet unknown, PSGL-1 ligands exist. The cytoplasmic signal transduction pathway of PSGL-1 is not obvious as the cytoplasmic tail of this receptor lacks classic signaling motifs. However, the cytoskeleton associated ezrin and moesin can serve as adaptors to ensue downstream activation of signaling cascades (45, 48, 49). In addition, localized receptor clustering may play a role as PSGL-1 is able to concentrate in the uropod region of polarized immune cells (45, 50). Further work is needed to define the immediate signaling pathways enabling the proinflammatory shift in macrophages induced by PSGL-1 blockade.

In this study, we identified the macrophage as an immune cell type with functional PSGL-1 expression across multiple types of human tumors. Consistent with previous studies investigating its role in T-cell inhibition (23, 45), our PSGL-1 siRNA knockdown and blocking antibody data indicate a novel, immunosuppressive signaling function for this adhesion molecule in human macrophages. Adhesion molecules are well suited to integrate spatial tissue information into the activation status of an immune cell (22, 45). PSGL-1 blockade resulted in decreased expression of M2 macrophage–associated markers CD163 and CD206. Furthermore, the PSGL-1 blocking mAb anti-PSGL-1 mediated proinflammatory cytokine release as well as release of T-cell recruiting chemokines. Importantly, the anti-PSGL-1–mediated macrophage repolarization effect was profound enough to elicit a proinflammatory response in patient-derived tumors in the presence of tumor cells and other inhibitory immune cell types. Even though we observed that macrophages are highly activated by exposure to PSGL-1, we cannot exclude that anti-PSGL-1 targets other PSGL-1 expressing immune cells in the tumor milieu, like T cells, Tregs or neutrophils, that may contribute to the antitumoral response. Because the human anti-PSGL-1 mAb lacks cross-reactivity to mouse PSGL-1, we generated a mouse surrogate anti-PSGL-1, which showed significant antitumor response as monotherapy and/or combination therapy with anti-PD-1 in multiple models. To further explore the effect of anti-PSGL-1 in a model system with more human relevant immune system, we analyzed the anti-PSGL-1–mediated immune effects in a humanized tumor-bearing mouse model. Anti-PSGL-1–induced TAM repolarization in concert with enhanced T-cell activation led to tumor growth inhibition. These findings are in line with previous experimental studies demonstrating reduced growth of murine tumors in PSGL-1–deficient mice as a result of increased T-cell activity (23, 25, 45). Notably, there is no depletion of T cells in anti-PSGL-1–treated tumors suggesting that PSGL-1 blockade does not impair T-cell migration.

Cancer therapies that entail a pharmacologic manipulation of TAMs are evaluated in early clinical trials utilizing various strategies (14, 51). Among these, the depletion of tumor promoting TAMs from the tumor bed and stimulating the tumor cell phagocytosing capability of TAM represent the clinically most advanced therapeutic concepts (14, 51, 52). Another promising TAM targeting strategy takes advantage of macrophages as potent effector cells, able to secrete a broad array of proinflammatory cytokines and chemokines, and hence setting off a cascade of secondary, adaptive immune responses (51). The first TAM repolarizing agents have entered the clinics in the form of anti-LILRB2 blocking antibodies (53). While there are multiple human LILRB family members, only a single mouse ortholog exists and is referred to as PIR-B (54). Comparable with PSGL-1–deficient mice, *Pirb*^−/−^ mice display an activated immune system and reduced tumor growth (54). However, in contrast to *Pirb*^−/−^, PSGL-1 deficiency led to spontaneous autoimmunity disease that showed exacerbated colitis upon dextran sodium sulfate challenge (47, 55). Furthermore, absence of PIR-B constitutively activated B cells via increased B-cell receptor signaling. Hence, it is tempting to speculate that targeting the APC-enriched LILRB2 may translate into a different clinical profile compared with the T cell–expressed PSGL-1 despite their shared functionality as molecular macrophage checkpoints.

It is important to note that PSGL-1 blocking antibodies require low-level stimulation (i.e., with LPS or SEB) to elicit cytokine release *in vitro*. For differentiation and sustained activation, macrophages rely on at least two independent signals for proinflammatory activation representing a safeguard mechanism to prevent excessive tissue damage (56). While a single signal, such as TLR activation can activate innate immune cells, it can activate monocytes or macrophages differentiated into either suppressive or inflammatory states. Because dying tumor cells in contrast to healthy tissue deliver damage-associated molecular patterns (57, 58) that serve as a second activating signal for a TAM repolarization agent, a decent therapeutic window can be expected.

Blockade of PSGL-1 offers the potential to provide clinical benefit to patients with cancer in monotherapy due to reprogramming the abundant macrophage infiltrate residing in the tumor bed and thus overcoming the local immunosuppressive milieu. Accordingly, our *ex vivo* treated patient-derived tumor cultures demonstrated antitumor immune responses and T-cell activation even in PD-1 mAb resistant tumors. Apart from cotargeting two immunosuppressive molecules on T cells that may cooperate, the release of T-cell recruiting chemokines such as CXCL9/10 and CCL-5 by TAMs could further bolster antitumor immunity. Arguably, high expression of these specific chemokines predicted responses to PD1/PD-L1 targeted therapies in patients and mouse models (59). In conclusion, therapeutic targeting PSGL-1 is a promising opportunity for the treatment of both T-cell immunotherapy resistant as well as responsive patients.

## Supplementary Material

Supplementary Figure 1 Single cell transcriptome atlas showing PSGL-1 expression on human immune cells in non-small cell lung cancer.

Supplementary Figure 2 All solid cancer indications in TCGA ranked by the average expression of SELPLG.

Supplementary Figure 3 Phenotype of M1, M0 and M2c macrophages.

Supplementary Figure 4 Characterization of Anti-PSGL-1.

Supplementary Figure 5 Anti-PSGL-1 repolarizes macrophages and leads to a pro-inflammatory response.

Supplementary Figure 6 Evaluation of activation conditions on multiple primary cells and cell lines.

Supplementary Figure 7 Anti-PSGL-1 induces pro-inflammatory response in a LPS stimulated mouse splenocytes.

Supplementary Figure 8 Induction of individual cytokines by anti-PSGL-1 treatment and Keytruda in fresh tumor cultures.

Supplementary Figure 9 Gating strategy for evaluating M1 and M2 macrophage and T cell activation markers in dissociated tumors from NGS-SGM3-BLT humanized mice.

Supplementary Table 1 Staining panels for the humanized mouse model experiment.

Supplementary Table 2 Lymphoid staining panel for the syngeneic Sa1N study.

Supplementary Table 3 Myeloid staining panel for the syngeneic Sa1N study.
