# Supplementary figures and images for: PSGL-1 Blockade Induces Classical Activation of Human Tumor-associated Macrophages
Source: Cancer Res Commun. 2023 Oct 26;3(10):2182–94. doi: 10.1158/2767-9764.CRC-22-0513 (PMC10601817; doi:10.1158/2767-9764.CRC-22-0513)

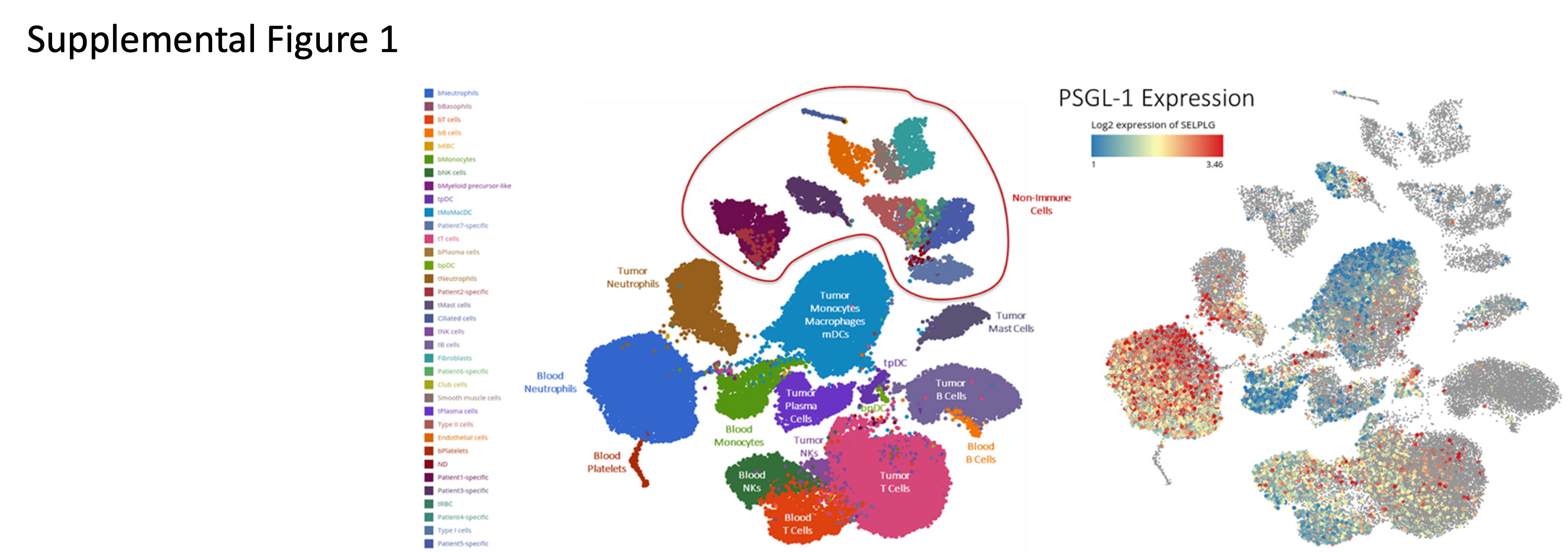

Supplement: Supplementary Figure 1 — Single cell transcriptome atlas showing PSGL-1 expression on human immune cells in non-small cell lung cancer. [file crc-22-0513-s01.png]

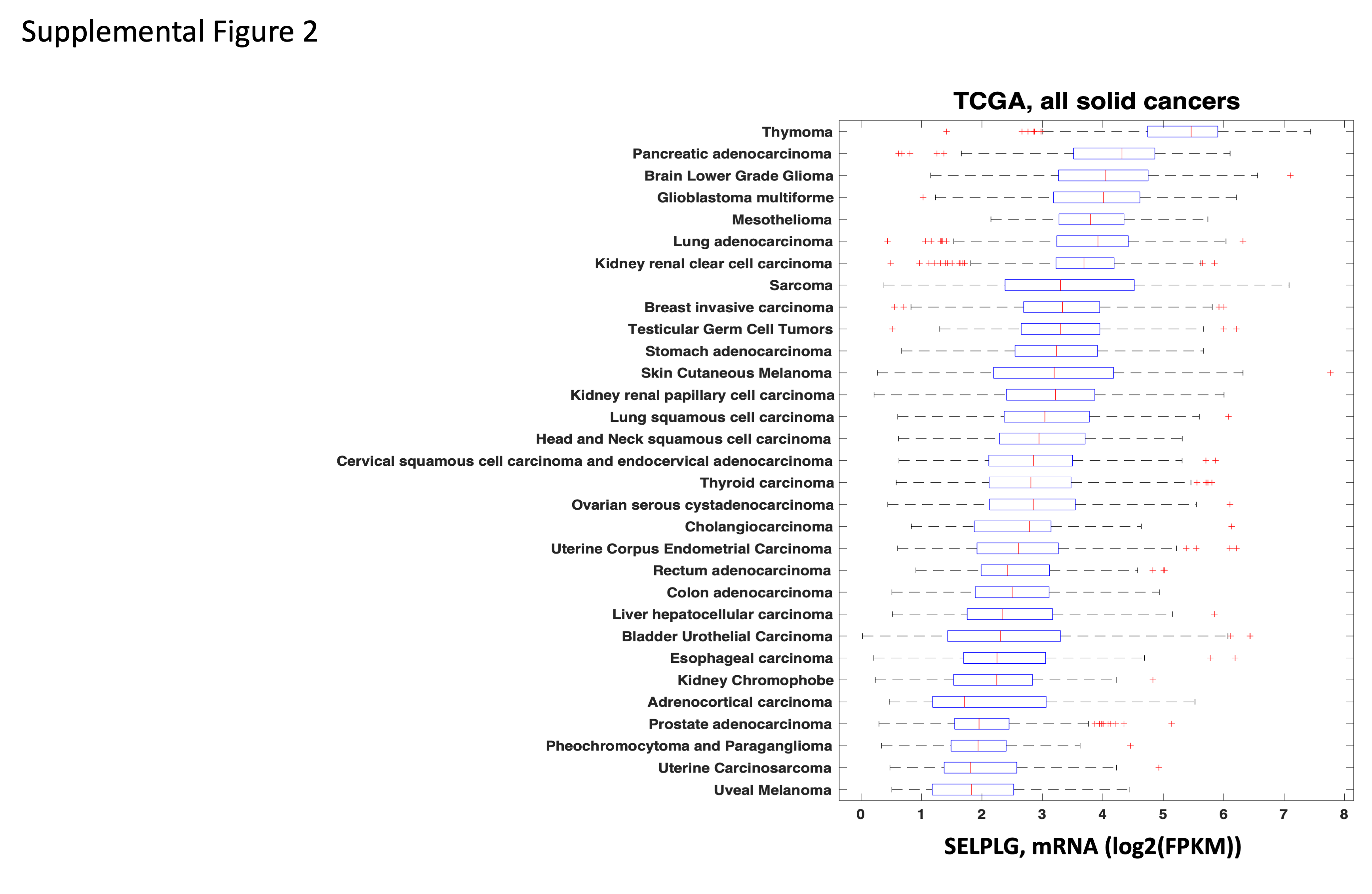

Supplement: Supplementary Figure 2 — All solid cancer indications in TCGA ranked by the average expression of SELPLG. [file crc-22-0513-s02.png]

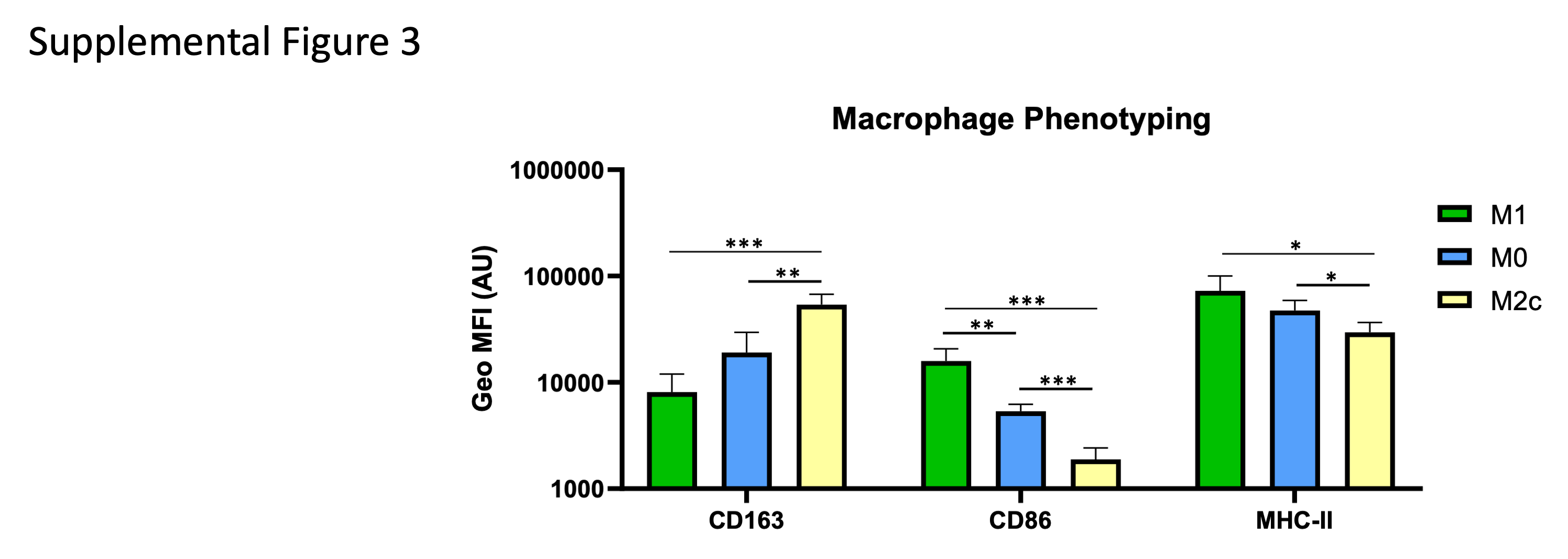

Supplement: Supplementary Figure 3 — Phenotype of M1, M0 and M2c macrophages. [file crc-22-0513-s03.png]

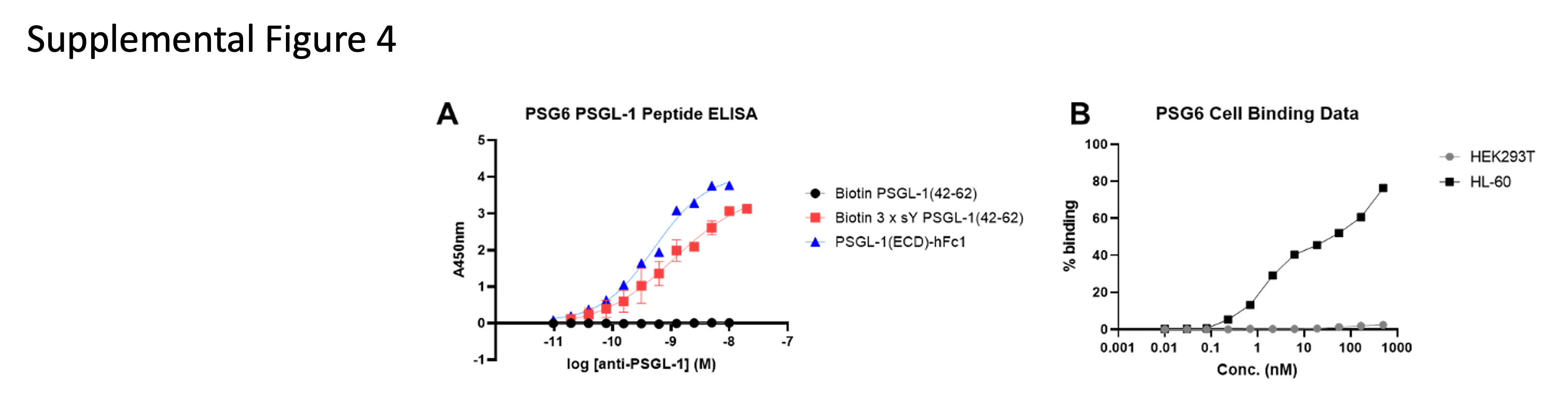

Supplement: Supplementary Figure 4 — Characterization of Anti-PSGL-1. [file crc-22-0513-s04.png]

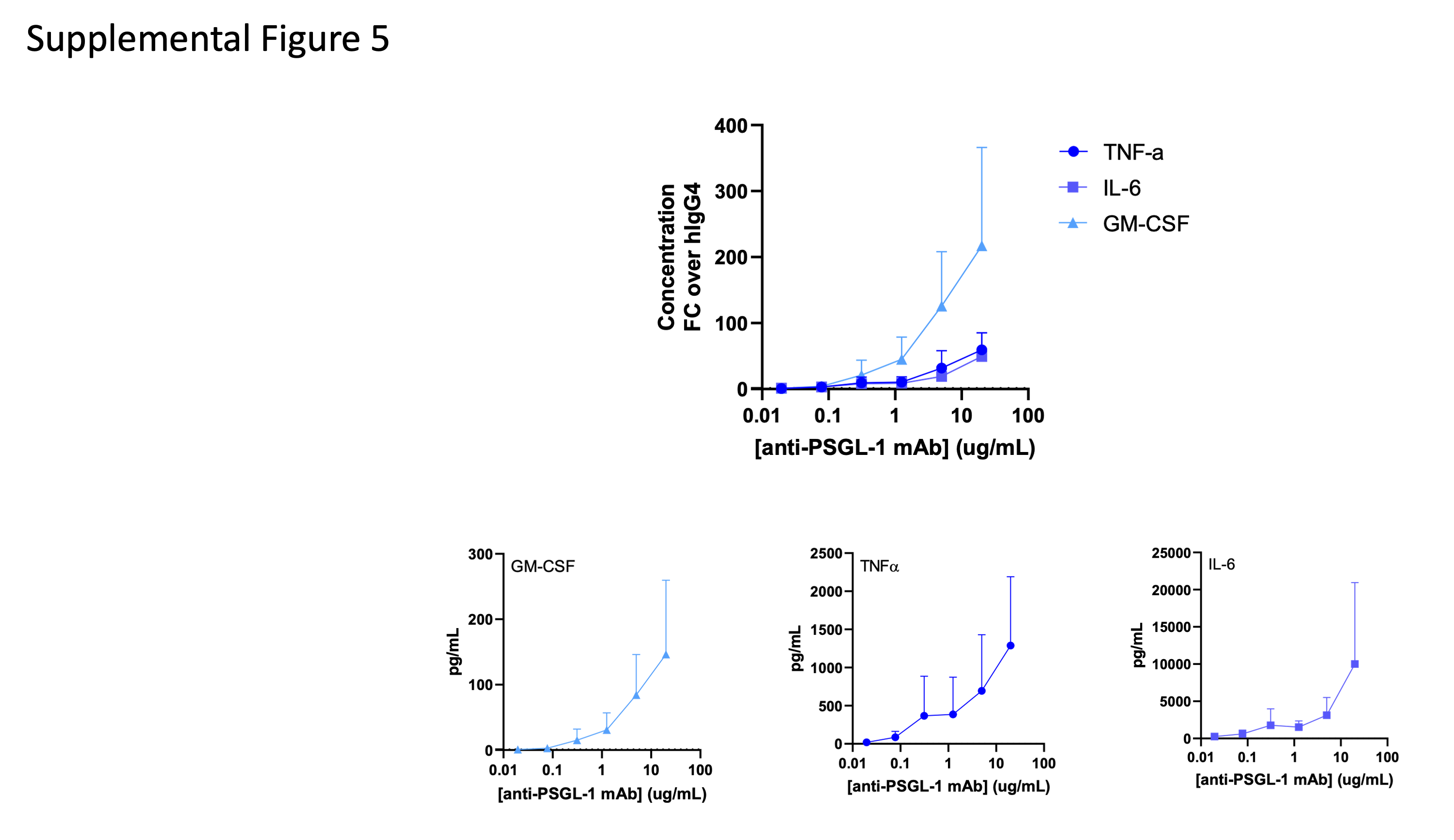

Supplement: Supplementary Figure 5 — Anti-PSGL-1 repolarizes macrophages and leads to a pro-inflammatory response. [file crc-22-0513-s05.png]

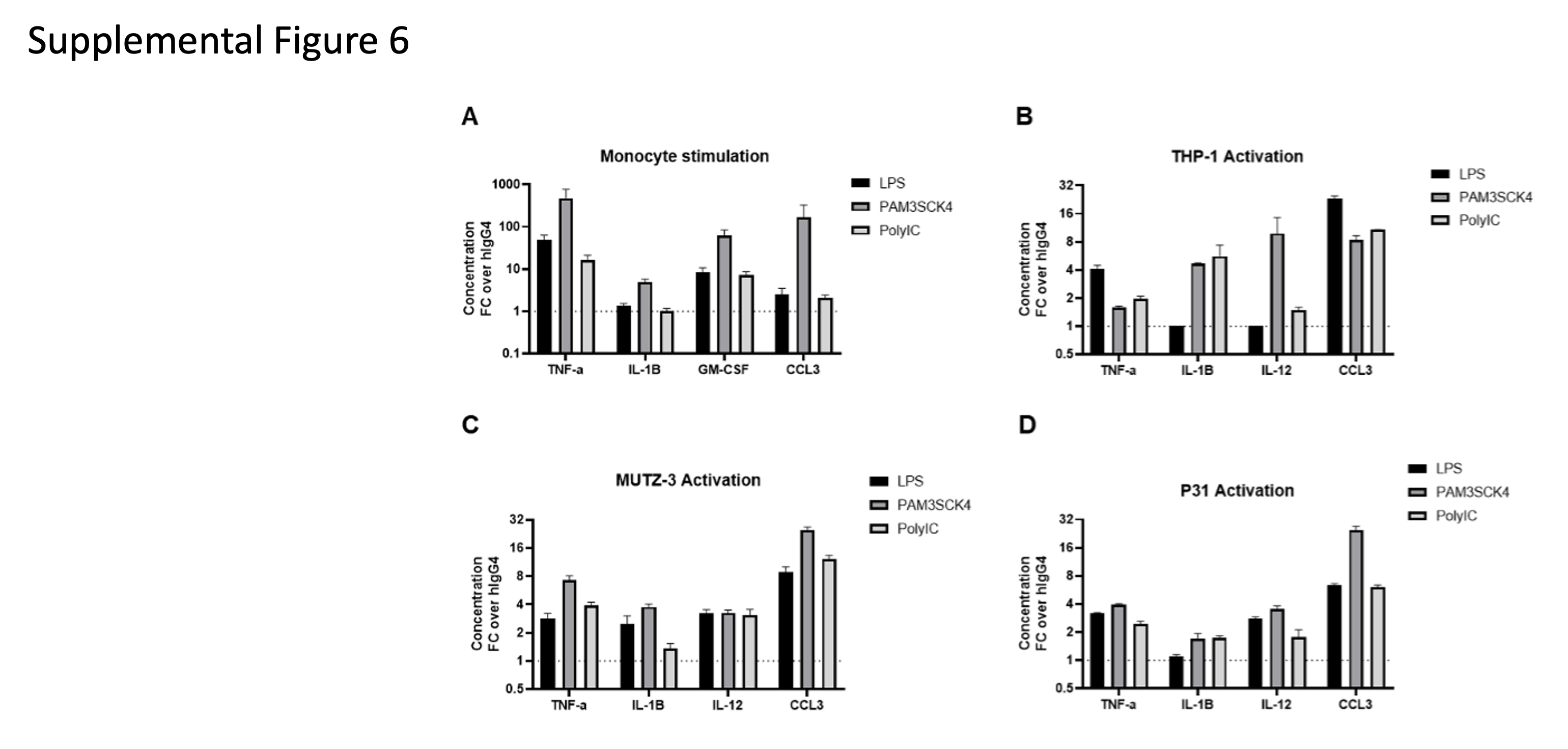

Supplement: Supplementary Figure 6 — Evaluation of activation conditions on multiple primary cells and cell lines. [file crc-22-0513-s06.png]

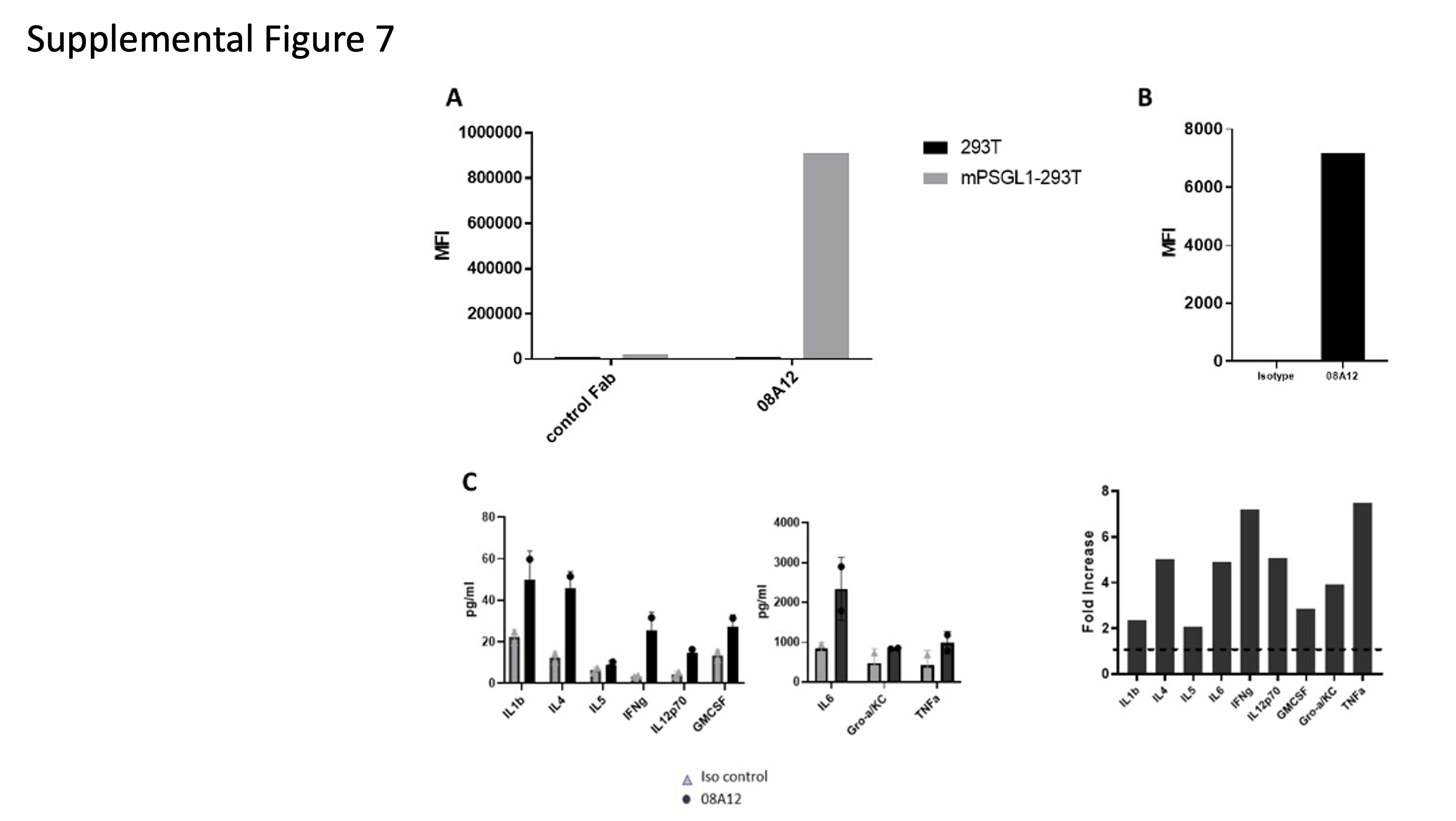

Supplement: Supplementary Figure 7 — Anti-PSGL-1 induces pro-inflammatory response in a LPS stimulated mouse splenocytes. [file crc-22-0513-s07.png]

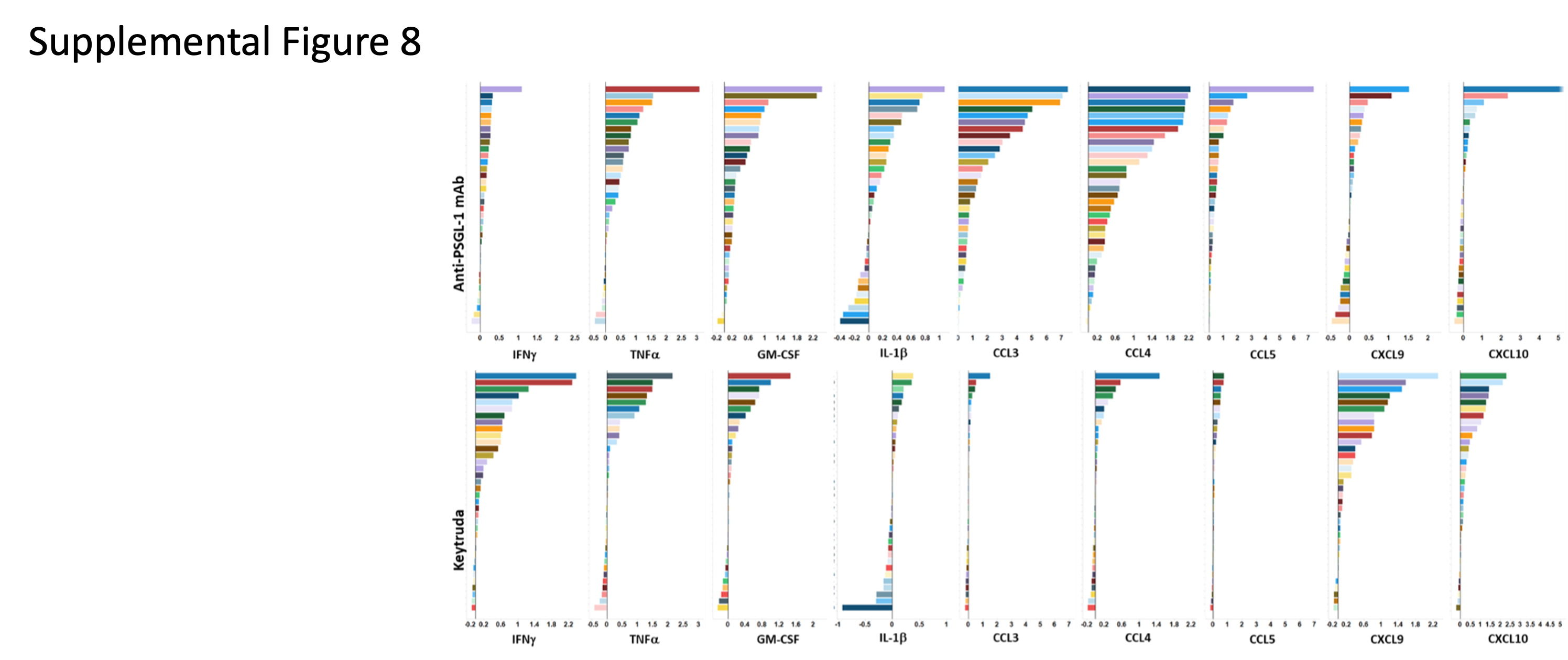

Supplement: Supplementary Figure 8 — Induction of individual cytokines by anti-PSGL-1 treatment and Keytruda in fresh tumor cultures. [file crc-22-0513-s08.png]

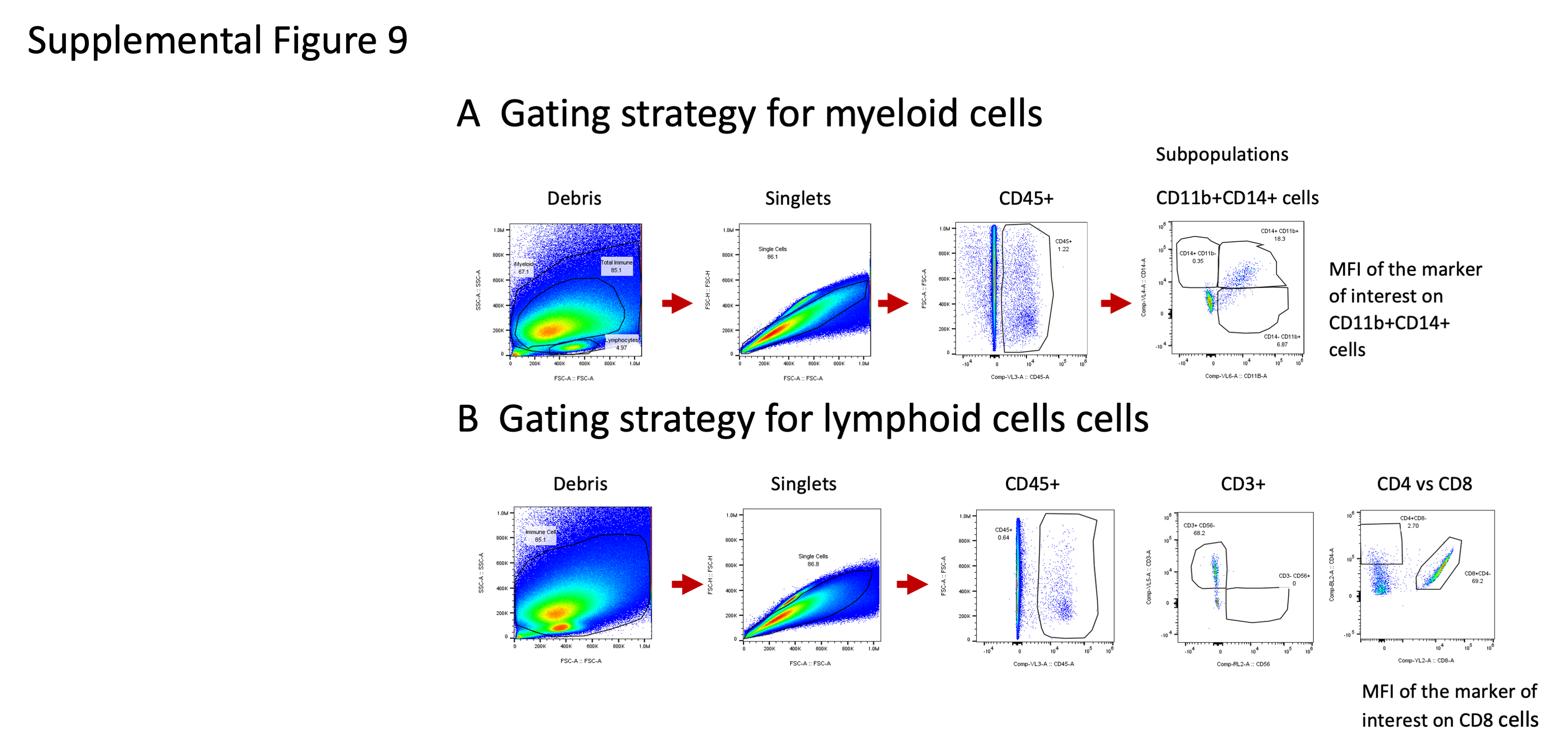

Supplement: Supplementary Figure 9 — Gating strategy for evaluating M1 and M2 macrophage and T cell activation markers in dissociated tumors from NGS-SGM3-BLT humanized mice. [file crc-22-0513-s09.png]

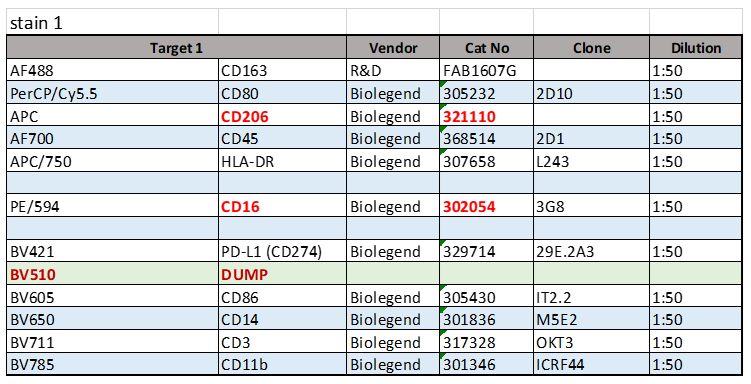


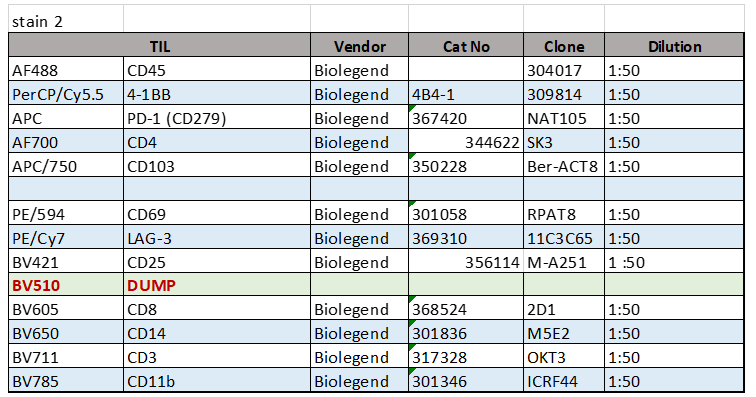


**Supplemental Table 1. Staining panels for the humanized mouse model experiment.**

Supplement: Supplementary Table 1 — Staining panels for the humanized mouse model experiment. [file crc-22-0513-s10.docx]
