# Supplementary material for: PSGL-1 Blockade Induces Classical Activation of Human Tumor-associated Macrophages
Source: Cancer Res Commun. 2023 Oct 26;3(10):2182–94. doi: 10.1158/2767-9764.CRC-22-0513 (PMC10601817; doi:10.1158/2767-9764.CRC-22-0513)
Supplement: Supplementary Table 2 — Lymphoid staining panel for the syngeneic Sa1N study. [file crc-22-0513-s11.docx]

| Conjugate | Antibody | Vendor | Cat # | Clone | Dilution |
| --- | --- | --- | --- | --- | --- |
| APC-eFluor 780 | Viability | ThermoFisher | 65-0865-14 | N/A | 1:500 |
| FITC | CD45 | ThermoFisher | 11-0451-82 | 30-F11 | 1:200 |
| PE-Cy7 | CD3 | BioLegend | 100220 | 17A2 | 1:100 |
| PerCP-Cy5.5 | CD4 | BioLegend | 100434 | GK1.5 | 1:200 |
| BV 510 | CD8 | BioLegend | 100752 | 53-6.7 | 1:250 |
| PE-Dazzle 594 | CD49b | BioLegend | 108924 | DX5 | 1:100 |
| PE-Dazzle 594 | NK1.1^*^ | BioLegend | 108748 | PK136 | 1:100 |
| eFluor 450 | GrB | ThermoFisher | 48-8898-82 | NGZB | 1:50 |
| BV 605 | CD25 | BioLegend | 102036 | PC61 | 1:100 |
| BV 711 | B220 | BioLegend | 103255 | RA3-6B2 | 1:66 |
| PE | FoxP3 | ThermoFisher | 12-5773-82 | FJK-16s | 1:50 |

**Supplemental Table 2. Lymphoid staining panel for the syngeneic Sa1N study.**
