# Supplementary material for: PSGL-1 Blockade Induces Classical Activation of Human Tumor-associated Macrophages
Source: Cancer Res Commun. 2023 Oct 26;3(10):2182–94. doi: 10.1158/2767-9764.CRC-22-0513 (PMC10601817; doi:10.1158/2767-9764.CRC-22-0513)
Supplement: Supplementary Table 3 — Myeloid staining panel for the syngeneic Sa1N study. [file crc-22-0513-s12.docx]

| Conjugate | Antibody | Vendor | Cat # | Clone | Dilution |
| --- | --- | --- | --- | --- | --- |
| APC-eFluor 780 | Viability | R&D Systems | ThermoFisher | 65-0865-14 | N/A |
| FITC | CD45 | Biolegend | ThermoFisher | 11-0451-82 | 30-F11 |
| PE-Cy7 | CD3 | Biolegend | BioLegend | 100220 | 17A2 |
| PerCP-Cy5.5 | Ly6-G | Biolegend | BioLegend | 127654 | 1A8 |
| BV 510 | Ly6-C | Invitrogen | BioLegend | 128033 | HK1.4 |
| PE-Dazzle 594 | CD206 | R&D Systems | BioLegend | 141732 | C068C2 |
| BV 605 | B220 | eBioscience | BioLegend | 103244 | RA3-6B2 |
| BV 711 | MHC II | Biolegend | BioLegend | 107643 | M5/114.15.2 |
| PE | CD163 | Biolegend | BioLegend | 155308 | S15049I |
| Alexa Fluor 700 | CD11b | Biolegend | BioLegend | 101222 | M1/70 |

**Supplemental Table 3. Myeloid staining panel for the syngeneic Sa1N study.**
